# Application of density functional theory for evaluating the mechanical properties and structural stability of dental implant materials

**DOI:** 10.1186/s12903-023-03691-8

**Published:** 2023-12-01

**Authors:** Ravinder Singh Saini, Seyed Ali Mosaddad, Artak Heboyan

**Affiliations:** 1 Department of Dental Technology, COAMS, King Khalid University, Abha, Saudi Arabia; 2 Student Research Committee, School of Dentistry, Shiraz University of Medical Sciences, Shiraz, Iran; 3 Department of Prosthodontics, Faculty of Stomatology, Yerevan State Medical University After Mkhitar Heratsi, Str. Koryun 2, 0025 Yerevan, Armenia

**Keywords:** DFT, Titanium, Zirconia

## Abstract

**Background:**

Titanium is a commonly used material for dental implants owing to its excellent biocompatibility, strength-to-weight ratio, corrosion resistance, lightweight nature, hypoallergenic properties, and ability to promote tissue adhesion. However, alternative materials, such as titanium alloys (Ti–Al-2 V) and zirconia, are available for dental implant applications. This study discusses the application of Density Functional Theory (DFT) in evaluating dental implant materials' mechanical properties and structural stability, with a specific focus on titanium (Ti) metal. It also discusses the electronic band structures, dynamic stability, and surface properties. Furthermore, it presents the mechanical properties of Ti metal, Ti–Al-2 V alloy, and zirconia, including the stiffness matrices, average properties, and elastic moduli. This research comprehensively studies Ti metal's mechanical properties, structural stability, and surface properties for dental implants.

**Methods:**

We used computational techniques, such as the CASTEP code based on DFT, GGA within the PBE scheme for evaluating electronic exchange–correlation energy, and the BFGS minimization scheme for geometry optimization. The results provide insights into the structural properties of Ti, Ti–Al-2 V, and zirconia, including their crystal structures, space groups, and atomic positions. Elastic properties, Fermi surface analysis, and phonon studies were conducted to evaluate the tensile strength, yield strength, ductility, elastic modulus, Poisson's ratio, hardness, fatigue resistance, and corrosion resistance.

**Results:**

The findings were compared with those of Ti–Al-2 V and zirconia to assess the advantages and limitations of each material for dental implant applications. This study demonstrates the application of DFT in evaluating dental implant materials, focusing on titanium, and provides valuable insights into their mechanical properties, structural stability, and surface characteristics.

**Conclusions:**

The findings of this study contribute to the understanding of dental implant material behavior and aid in the design of improved materials with long-term biocompatibility and stability in the oral environment.

**Supplementary Information:**

The online version contains supplementary material available at 10.1186/s12903-023-03691-8.

## Background

Titanium is widely used in dentistry for dental implants as synthetic tooth roots that offer stability and support for prosthetic teeth [1–3]. Titanium is an excellent choice for dental implants for several reasons. It has excellent biocompatibility, meaning the human body tolerates it well and fuses with the jawbone through osseointegration, ensuring implant stability [1–3]. Titanium is known for its exceptional strength-to-weight ratio, making it resistant to fractures and corrosion. This allows titanium implants to withstand chewing forces and provide long-lasting support for replacement teeth. Titanium is lightweight, reducing strain on the jawbone and promoting patient comfort while minimizing the risk of bone loss. It is hypoallergenic and rarely causes allergic reactions, making it suitable for a wide range of individuals, including those with metal sensitivity. Titanium implants have a smooth surface that promotes tissue adhesion and prevents bacterial growth, reducing the risk of infection and inflammation [4–6]. They have a high success rate and can last decades with proper maintenance, improving chewing performance and esthetics. While titanium is commonly used, alternative materials like titanium alloys and zirconia are available, which may depend on individual needs and recommendations from the dentist [7–11].

Advancements in dental implant technology have revolutionized dental restorative procedures, offering patients a reliable and aesthetically pleasing solution for replacing missing teeth. As the demand for dental implants grows, there is an increasing need for materials that provide excellent mechanical performance and ensure long-term biocompatibility and stability within the oral environment. Traditional empirical approaches to material evaluation are limited in understanding the intricate atomic and electronic interactions that govern material behavior. Conventional approaches to evaluating dental implant materials have limitations in providing a comprehensive understanding of material behavior, especially concerning intricate atomic and electronic interactions. These methods often fail to capture the detailed electronic structure of materials, making it challenging to accurately study bonding mechanisms, charge transfer phenomena, and the impact of defects or impurities. As a result, the development of dental implant materials that offer both excellent mechanical performance and long-term biocompatibility within the oral environment is hindered. Density Functional Theory (DFT) emerges as a groundbreaking solution to overcome these limitations. DFT is a powerful and versatile computational tool that allows researchers to investigate materials at the quantum level. Unlike traditional methods, DFT provides a detailed description of the electronic structure of materials. It evaluates the distribution of electrons within dental implant materials, enabling a deep understanding of atomic and electronic interactions. Researchers can delve into bonding mechanisms, explore charge transfer phenomena, and analyze the effects of defects or impurities with remarkable precision. By harnessing the capabilities of DFT, scientists and engineers can design dental implant materials with improved mechanical strength, enhanced biocompatibility, and reduced susceptibility to degradation. This shift from traditional empirical approaches to the advanced capabilities of DFT marks a significant stride in developing dental implant technology, ensuring that the materials used meet the stringent requirements of durability, stability, and biocompatibility essential for successful dental restorative procedures. Density Functional Theory (DFT) bridges this gap by offering a powerful and versatile tool for accurately and systematically investigating dental implant materials at the quantum level [12, 13]. One of the key advantages of employing DFT is its ability to provide a detailed description of the electronic structure of materials. By evaluating the distribution of electrons within dental implant materials, DFT enables researchers to study bonding mechanisms, charge transfer phenomena, and the influence of defects or impurities on material properties. Such insights can be pivotal in designing dental implant materials with improved mechanical strength, enhanced biocompatibility, and reduced susceptibility to degradation [14, 15].

Furthermore, DFT allows the exploration of a wide range of material configurations and compositions, enabling researchers to screen numerous potential dental implant materials in silico. This significantly reduces the need for time-consuming and costly experimental trials and accelerates the discovery of novel materials with optimized mechanical properties and structural stability. As dental implant materials are exposed to varying oral conditions and mechanical stresses, accurately predicting their response to different environments is paramount. Using DFT, researchers can simulate the behavior of dental implant materials under different physiological conditions to aid in understanding how the materials may degrade or undergo phase transitions over time, thus guiding the selection of materials that can withstand the challenges posed by the oral environment [16, 17].

Moreover, DFT can provide valuable insights into the mechanical behavior of dental implant materials under dynamic loading conditions. By studying these materials' elastic properties, deformation mechanisms, and fracture behavior, researchers can assess their long-term structural stability and resistance to fatigue failure as critical factors for ensuring the success and longevity of dental implants [18–20].

In this article, we conducted a comprehensive study on Ti metal's mechanical properties, structural stability, and surface properties for dental implants. We investigated these aspects through the analysis of elastic properties, Fermi surface analysis, and phonon studies. Furthermore, we compared these findings with those of Ti–Al-2 V and zirconia, alternative materials commonly used in dental implant applications. Regarding the mechanical properties, we examined Ti metal's tensile strength, yield strength, and ductility. We gained insights into the material's ability to withstand forces and its deformation behavior by evaluating its elastic modulus, Poisson's ratio, and hardness. We also explored the fatigue resistance and corrosion resistance of Ti metal, which are crucial factors for long-term durability in dental implant applications. By comparing the findings with those of Ti–Al-2 V and zirconia, we evaluated the advantages and limitations of each material for dental implant applications. This comparative analysis has provided valuable insights into the suitability, performance, and potential challenges associated with using Ti metal compared to alternative materials.

## Methods

Cambridge Serial Total Energy Package (CASTEP) code was used, specifically within Material Studio 2020, along with ELATE for elastic tensor analysis, to generate the data and graphs presented in our study. The ground-state energy of the material was calculated using the Cambridge Serial Total Energy Package (CASTEP) code [21, 22], which employs a first-principles technique based on density functional theory (DFT) [23–25]. The electronic exchange–correlation energy was evaluated using the generalized gradient approximation (GGA) within the Perdew-Burke-Ernzerhof (PBE) scheme [26]. To represent the interaction between the valence electrons and ion cores of atoms, Vanderbilt-type ultra-soft pseudopotentials were utilized [27, 28]—This choice of pseudopotential balances computational efficiency and accuracy. The valence electron configurations considered were 3s^2^ 3p^6^ 3d^2^ 4s^2^ for the Ti atoms.

Geometry optimization of Ti was performed using the Limited-memory Broyden–Fletcher–Goldfarb–Shanno (LBFGS) minimization scheme [29] to obtain the lowest energy structure. A plane-wave cutoff energy of 500 eV (for Ti–Al-2 V 280 eV and Zirconia 300 eV) was used for the expansion. Brillouin zone (BZ) integrations were carried out employing the Monkhorst–Pack method [2, 30] with a 20 × 20 × 11 special k-point mesh (for Ti–Al-2 V 1 × 1 × 1 and zirconia 3 × 3 × 1). Additionally, Fermi surfaces were obtained by sampling the entire BZ using a 35 × 35 × 35 k-point mesh. Geometry optimization was conducted with convergence tolerances of 10^–4^ eV/atom for total energy, 10^–2^ Å for maximum lattice point displacement, 0.03 eV Å^−1^ for maximum ionic Hellmann–Feynman force, and 0.05 GPa for maximum stress tolerance. Finite basis-set corrections were applied [31]. These tolerance levels ensured reliable estimations of the structural, elastic, and electronic band structure properties while maintaining computational efficiency.

## Results and discussion

### Structural properties

**Fig. 1:**
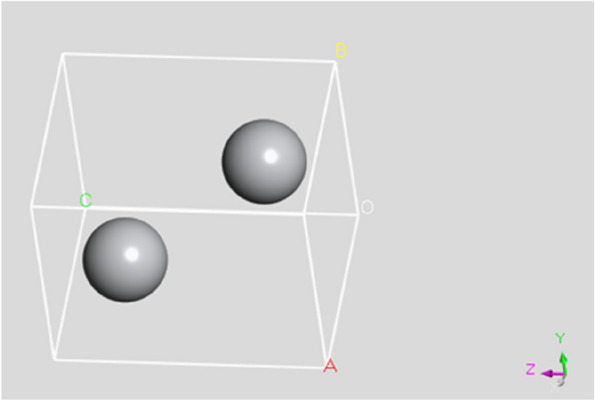
3D Crystal structure of the Ti unit cell

Ti (titanium) assumes a simple hexagonal crystal structure with the space group *P*63/MMC (space no. 194). In this crystal structure, the Ti atoms are positioned at simple cubic corner lattice points, as depicted in Fig. 1. The hexagonal structure of Ti is characterized by a close-packed arrangement of atoms along the c-axis and a hexagonal lattice in the basal plane. The Ti atoms occupy a primitive-centered position within the unit cell at coordinates (0, 0, 0) [32, 33]. This implies that the Ti atoms are at the center of the hexagonal unit cell, contributing to its structural stability and symmetry. Ti's simple hexagonal crystal structure is essential in determining its physical and chemical properties. The arrangement of atoms in this structure affects properties such as mechanical strength, electrical conductivity, and thermal behavior. Crystallographic details, such as the space group and atomic positions, provide valuable information for understanding the crystal symmetry, crystallographic planes, and directions in Ti.

**Fig. 2:**
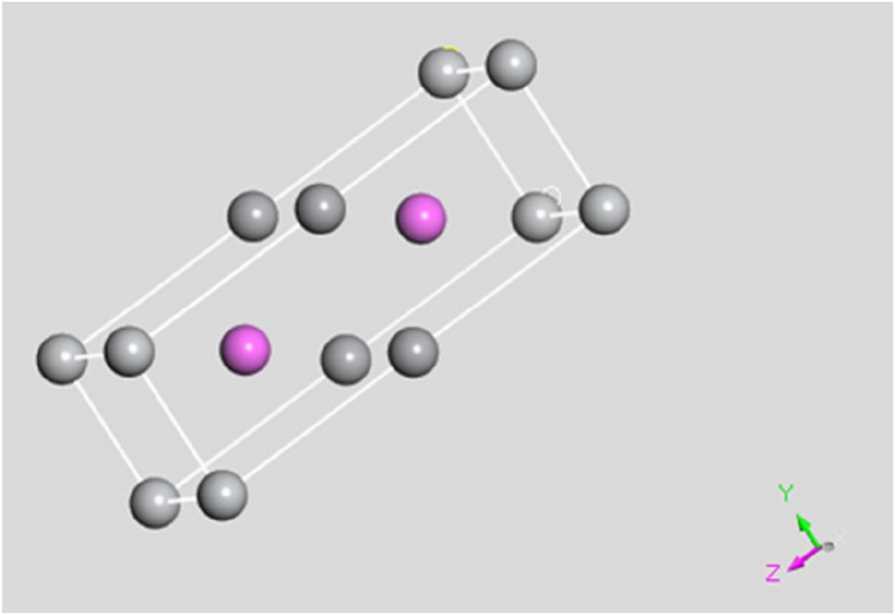
3D crystal structure of the Ti–Al-2 V unit cell

Ti–Al-2 V, a captivating alloy, exhibits a unique crystal structure that belongs to a simple triclinic crystal system. This crystal structure is characterized by space group P1 (space no. 1), highlighting the alloy's intricate arrangement and ability to exhibit a wide range of properties [34]. Within this fascinating structure, titanium (Ti) atoms assume positions at the simple cubic corner lattice points, adding stability and symmetry to the overall arrangement. Expanding our exploration, we discovered that the arrangement of atoms within Ti–Al-2 V extends beyond the corner lattice points. At the heart of the crystal, each aluminum (Al) atom gracefully resides at the center, acting as a pivotal anchor within the structure. Meanwhile, vanadium (V) atoms find their place between the titanium (Ti) atoms along the c-axis, harmoniously filling the spaces and enhancing the overall arrangement. Figure 2 captures this intricate distribution, visually representing the precise positioning of atoms within the Ti–Al-2 V crystal lattice.

**Fig. 3:**
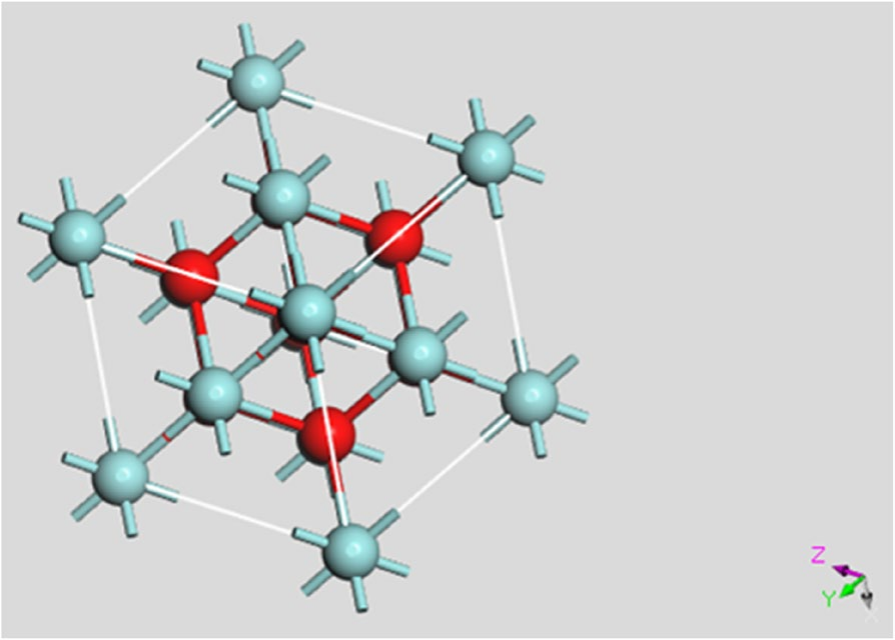
3D crystal structure of zirconia unit cell

Zirconia exhibits a remarkable crystal structure characterized by a simple cubic arrangement. This crystal system adopts the space group FM-3 M (space no. 192), which reveals its inherent symmetry and organization [35]. In this captivating arrangement, Zn atoms elegantly occupy simple cubic corner lattice points, gracefully positioned at coordinates (0,0,0), as if carefully orchestrated by nature itself. Delving deeper into the mesmerizing structure, the Zn atoms at the corner lattice points extend their influence to the heart of the crystal. Each Zn atom forms a vital connection, reaching an O atom positioned meticulously in the middle. This intricate interaction between the Zn and O atoms can be vividly visualized in the illustrative depiction provided in Fig. 3, offering a glimpse into the harmonious dance of atoms within the zirconia lattice.

## Electronic band structure and dynamic stability

The band structure calculation provides information about the electronic energy levels of the titanium implant along different high-symmetry directions in the first Brillouin zone (BZ). Moreover, the energy range of the phonon structure, ranging from -20 to 80 eV, represents the energy of the lattice vibrations (phonons) [36] in the titanium implant (Fig. 3). The presence of phonon modes within this energy range indicates the crystal lattice's vibrational degrees of freedom and stability. Additionally, to assess the dynamic stability of titanium implants, it is necessary to examine the phonon dispersion relations and the presence or absence of imaginary frequencies within the calculated energy range. Imaginary frequencies in the phonon spectrum indicate the presence of stable modes and suggest that the crystal lattice is dynamically stable (the allowed negative frequency is observed). The dynamic stability of titanium implants is crucial for their long-term performance and reliability. A dynamically stable implant ensures that the crystal lattice remains intact, preventing the occurrence of structural instabilities, phase transitions, or lattice defects that could compromise the mechanical integrity and functionality of the implant.

**Fig. 4:**
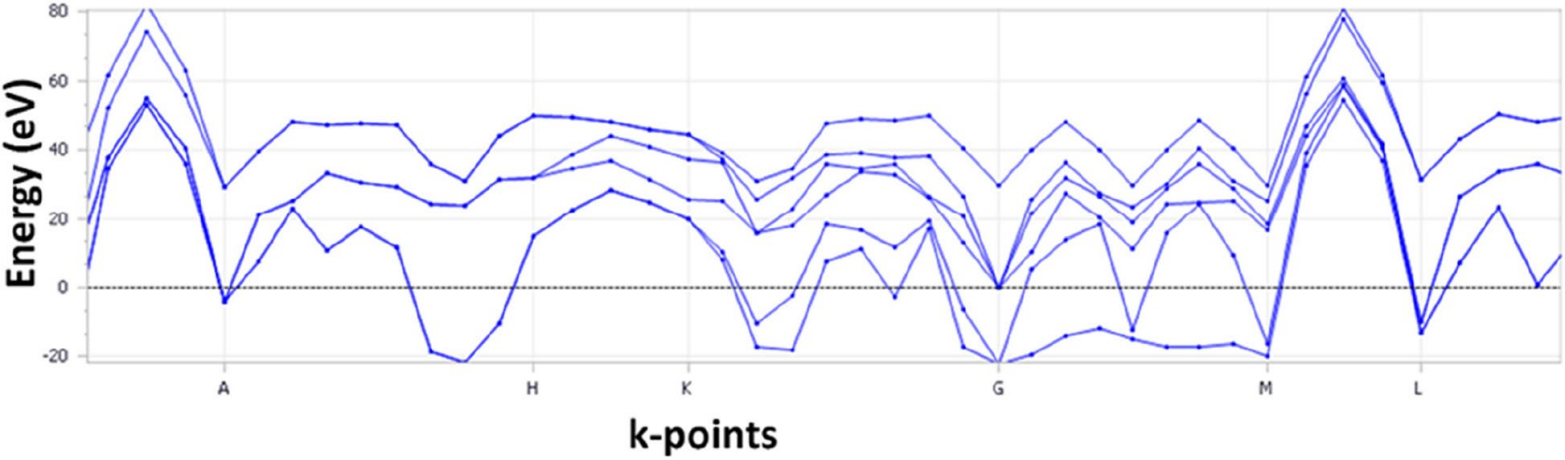
Electronic band structure of the Ti unit cell

It is important to note that the dynamic stability analysis based on the phonon structure provides information about the lattice vibrations and the absence of imaginary frequencies within the specified energy range. However, a comprehensive assessment of the implant's stability would require considering additional factors such as temperature, pressure, surface effects, and interaction with surrounding tissues (Fig. 4).

**Fig. 5:**
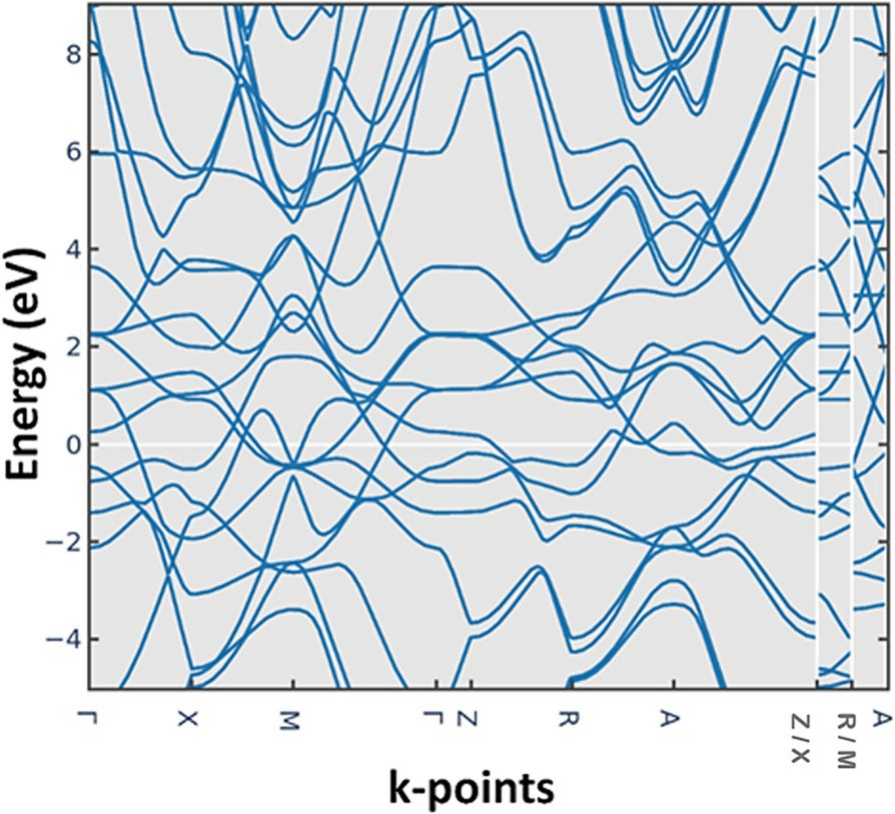
Electronic band structure of the Ti–Al-2 V alloy

The absence of any bands between the energy bands along different high-symmetry directions (Γ-X-M-Z-R-A-Γ) indicates a complete bandgap in the electronic band structure of the Ti–Al-2 V implant (Fig. 5). The energy range of the phonon structure for Ti–Al-2 V is -4 to 8 eV, representing the energy of the implant's lattice vibrations (phonons). Analyzing the presence or absence of imaginary frequencies within this range is crucial to assessing dynamic stability. To evaluate the dynamic stability of Ti–Al-2 V, we examined the phonon dispersion relations and the presence of imaginary frequencies. The imaginary frequencies in the phonon spectrum indicate unstable modes and a dynamically unstable crystal lattice. To compare the dynamic stability of Ti–Al-2 V with titanium, titanium is relatively more stable than Ti–Al-2 V.

**Fig. 6:**
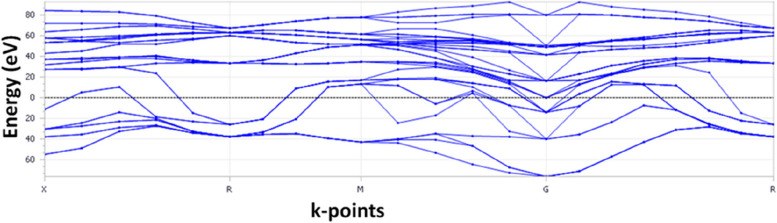
Electronic band structure of zirconia

The absence of any bands between the energy bands along different high-symmetry directions (Γ-X-R-M-G-R-Γ) indicates a complete bandgap in the electronic band structure of zirconia. This suggests that zirconia behaves as an insulator or semiconductor rather than a metal, similar to titanium. The energy range of the phonon structure for zirconia is -60 to 80 eV, representing the energy of the material's lattice vibrations (phonons) (Fig. 6). The presence of imaginary frequencies in the phonon spectrum indicates the presence of unstable modes and suggests a dynamically unstable crystal lattice. Comparing the given phonon energy range for zirconia (-60 to 80 eV) with that for titanium (-20 to 80 eV), it can be observed that the energy range for zirconia is significantly wider. This broader range suggests that zirconia may exhibit a higher vibrational energy range and potentially a more complex phonon structure than titanium. In summary, we can infer that zirconia may display a different dynamic stability profile than titanium owing to its wider energy range for the phonon structure.

## Surface properties and stability

**Fig. 7:**
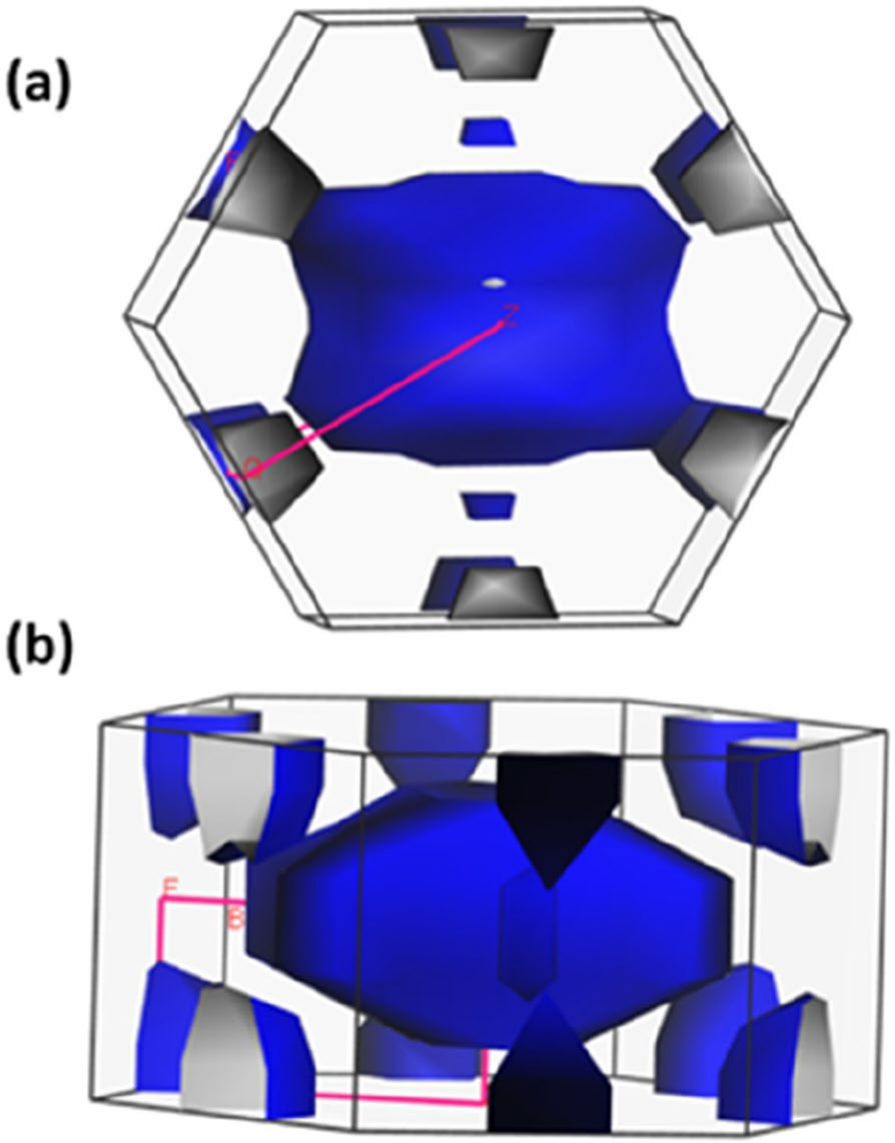
Fermi-surface (**a**) front view (**b**) side view of the Ti dental implant

A well-defined and nearly complete drum-shaped Fermi surface (Fig. 7) suggests Ti has good mechanical stability. A complete Fermi surface indicates a stable electronic structure and a well-organized arrangement of atoms within the crystal lattice. The nearly closed nature of the Fermi surface implies strong interatomic bonding within the Ti crystal. This is important for the structural integrity of titanium implants as it ensures good stability and resistance to deformation or failure. Ti is known for its excellent biocompatibility, and the properties reflected in its Fermi surface support this characteristic. A well-defined Fermi surface indicates a well-ordered electronic structure less likely to induce adverse reactions when in contact with biological tissues. Titanium implants have been widely used in orthopedics and dentistry owing to their ability to integrate with bone tissue. The properties reflected on the Fermi surface, such as good mechanical stability and interatomic bonding, suggest that titanium implants may facilitate osseointegration, forming a solid bond between the implant and the surrounding bone. Fermi surface refers to the surface in the momentum space of a material's electrons at absolute zero temperature. In the case of titanium, the properties observed on its Fermi surface, such as good mechanical stability and interatomic bonding, have implications for its use in dental implants. Based on these properties reflected on the Fermi surface, it is suggested that titanium implants can facilitate osseointegration. Titanium's good mechanical stability and interatomic bonding enhance its ability to integrate with the bone tissue, forming a solid bond between the implant and the surrounding bone. It is important to note that while Fermi surface analysis provides insights into titanium's electronic structure and specific surface properties, other factors such as surface topography, surface chemistry, and surface coatings can also influence the behavior and performance of titanium implants.

## Mechanical properties of Ti metal

### Stiffness matrix

To determine the suitability of titanium for dental implants based on the given stiffness matrix, we need to analyze its mechanical properties. The stiffness matrix provides information regarding the material's response to external forces and its ability to resist deformation. In this case, the coefficients in the stiffness matrix are expressed in gigapascals (GPa), which are units of pressure (Table 1). The stiffness matrix represents the material's response to the stress and strain. Each element in the matrix corresponds to a specific deformation direction. The diagonal elements represent the resistance of the material to stretching or compression along the respective axes, whereas the off-diagonal elements represent the resistance of the material to shear deformation. Based on the given stiffness matrix, we can observe that the diagonal elements (E11, E22, E33) are relatively high, indicating a high modulus of elasticity in the longitudinal directions. This suggests that Ti exhibits excellent resistance to deformation when subjected to axial forces. This is desirable for dental implants, as they need to withstand chewing forces. The off-diagonal elements (E12, E13, and E23) are also relatively high but slightly lower than the diagonal elements. This indicates good resistance to shear deformation. Shear forces can occur during biting or grinding motions, and the high values suggest Ti can withstand these forces well. The non-zero elements outside the diagonal (E14, E24, E34, E45, and E56) represent the coupling effects between the different directions of deformation. These coefficients were all zero in the given matrix, suggesting that titanium did not exhibit significant coupling effects in the context of the provided data. Overall, the stiffness matrix indicates that titanium has favorable mechanical properties for dental implants. It demonstrates high stiffness and resistance to deformation along the longitudinal and shear directions, which are crucial for withstanding the forces experienced in the oral cavity. However, it is essential to note that other factors, such as biocompatibility, corrosion resistance, and osseointegration, also play significant roles in determining the suitability of titanium for dental implant applications.

**Table 1:** Stiffness matrix (coefficients in GPa) of Ti metal

121.06	94.09	78.486	0	02	03
94.09	121.06	78.486	0	0	0
78.486	78.486	188.48	0	0	0
0	0	0	44.526	0	0
0	0	0	0	44.526	0
0	0	0	0	0	13.485

## Average properties

To assess the suitability of titanium for dental implants based on the average properties provided, several key mechanical parameters must be considered: bulk modulus, Young's modulus, shear modulus, and Poisson's ratio. These properties determine the response of Ti to external forces and its ability to resist deformation. Table 2 presents three averaging schemes: Voigt, Reuss, and Hill. Each scheme provides an average value for the properties based on different assumptions. The Voigt average assumes that the material behaves as if it is perfectly rigid in some directions and compliant in others. This average represents the upper bound of the properties of the composite material. The Reuss average assumes that material behaves as if it is perfectly compliant in some directions and perfectly rigid in others. This average represents the lower bound of the properties of the composite material. The Hill average represents the mean value between the Voigt and Reuss averages. It assumes a combination of rigid and compliant behaviors in different directions.

**Table 2:** Average properties of Ti metal

Averaging scheme	Bulk modulus	Young's modulus	Shear modulus	Poisson's ratio
Voigt	*K*_V_ = 103.64 GPa	*E*_V_ = 88.215 GPa	*G*_V_ = 32.477 GPa	*ν*_V_ = 0.35813
Reuss	*K*_R_ = 101.49 GPa	*E*_R_ = 64.744 GPa	*G*_R_ = 23.228 GPa	*ν*_R_ = 0.39368
Hill	*K*_H_ = 102.56 GPa	*E*_H_ = 76.621 GPa	*G*_H_ = 27.852 GPa	*ν*_H_ = 0.37549

Nevertheless, the bulk modulus was approximately 101–104 GPa, indicating that titanium has good resistance to compression. This property is crucial for withstanding the forces exerted during chewing. The Young's modulus ranges from 64–88 GPa, reflecting the material's stiffness. A higher Young's modulus suggests better resistance to deformation, which is favorable for dental implants. The shear modulus ranges from 23–32 GPa, indicating the material's resistance to shear deformation. Higher shear modulus values imply better resistance to forces that cause sliding or twisting. The Poisson's ratio ranges from 0.358–0.394, representing the ratio of lateral to axial strain. These values suggest titanium exhibits relatively low lateral expansion when subjected to axial forces. Overall, Ti demonstrated favorable average properties for dental implant applications. It exhibits high stiffness, good resistance to deformation, and suitable shear resistance. However, it is essential to consider other factors, such as biocompatibility, corrosion resistance, and osseointegration, when evaluating the suitability of titanium for dental implants.

To further evaluate the suitability of titanium for dental implants, we analyzed the significance of eigenvalues of the stiffness matrix values. Eigenvalues represent the characteristic values of the stiffness matrix, indicating the material's response to different modes of deformation. The eigenvalues are listed in Table 3.

**Table 3:** Eigenvalues of stiffness matrix of Ti metal

λ_1_(Gpa)	λ_1_ (Gpa)	λ_1_ (Gpa)	λ_1_ (Gpa)	λ_1_ (Gpa)	λ_1_ (Gpa)
13.485	26.969	44.526	44.526	90.022	313.61

The eigenvalues λ1, λ2, and λ3 (13.485 GPa, 26.969 GPa, and 44.526 GPa, respectively) represent the material's response to stretching or compression along different axes. These values indicate the stiffness of the material in these directions. Higher eigenvalues imply a higher resistance to deformation, suggesting that titanium has good stiffness along these axes. The eigenvalues λ4 (44.526 GPa) indicate the material's response to shear deformation. A higher eigenvalue for shear deformation indicates greater resistance to shearing forces. In this case, titanium exhibited a relatively high eigenvalue, suggesting good shear resistance. The eigenvalues λ5 (90.022 GPa) and λ6 (313.61 GPa) represent the material's response to complex deformation modes involving multiple directions. These values indicate the material's overall stiffness in response to the combined loading conditions. Higher eigenvalues indicate greater overall stiffness and resistance to deformation.

Based on the eigenvalues, titanium demonstrates favorable stiffness properties for dental implants. It exhibited high eigenvalues across different deformation modes, indicating good resistance to stretching, compression, and shear deformation. This suggests that titanium is well suited for withstanding the forces exerted on dental implants during chewing and other oral activities.

## Elastic moduli of Ti metal

To evaluate the suitability of titanium for dental implants based on the variations in the elastic moduli, we considered the range and anisotropy of the material properties. Table 4 provides information on Young's modulus, linear compressibility, shear modulus, Poisson's ratio, and their minimum and maximum values.

**Table 4:** Variations in the elastic moduli of Ti metal

	**Young's modulus**		**Linear compressibility**		**Shear modulus**		**Poisson's ratio**		
	***E*** **min**	***E*** **max**	**β** **min**	**β** **max**	***G*** **min**	***G*** **max**	**ν** **min**	**ν** **max**	
Value	45.709 GPa	131.22 GPa	2.0607 TPa^–1^	3.8962 TPa^–1^	13.485 GPa	44.526 GPa	-0.032242	0.77892	Value
Anisotropy	2.871		1.8907		3.302		∞		Anisotropy
Axis	1	0	0	1	0.7304	0	0.6945	0.1345	Axis
	0	0	0	0	0.683	0	-0.0002	0.7409	
	0	1	1	0	0	1	0.7195	-0.658	
					-0.683	0.766	0.7195	-0.9839	Second axis
					0.7304	0.6428	-0.0004	0.1786	
					0	0	-0.6945	0	

**Fig. 8:**
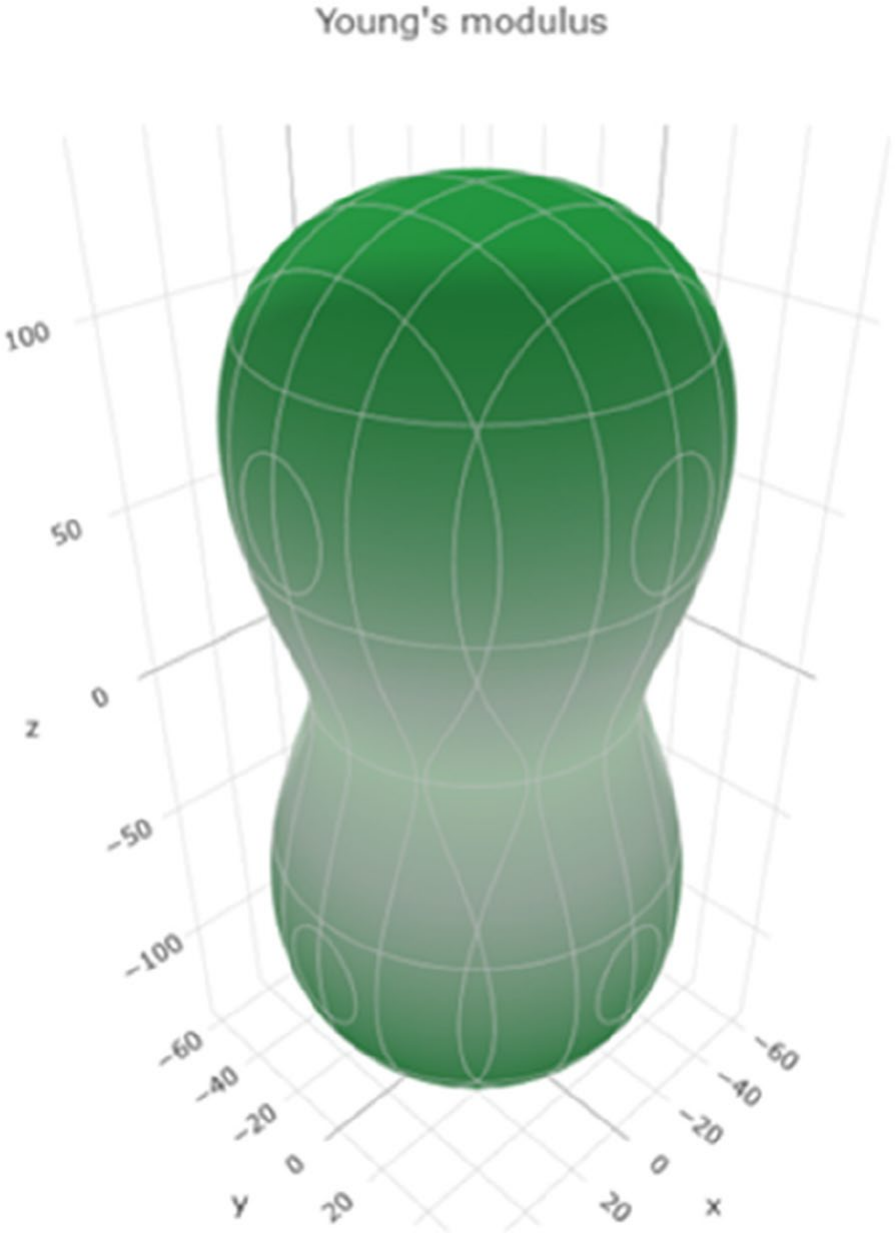
3D representation of the Young's modulus of Ti metal

Young's modulus represents the material's stiffness and its resistance to deformation under tensile or compressive forces. Figure 8 shows a 3D representation of Young's modulus, and Figure S1 shows a 2D representation of Young's modulus in the xy, xz, and yz planes. The range of values suggests that titanium can exhibit a wide range of stiffnesses depending on the specific conditions. Higher Young's motheus values indicate greater stiffness and resistance to deformation.

**Fig. 9:**
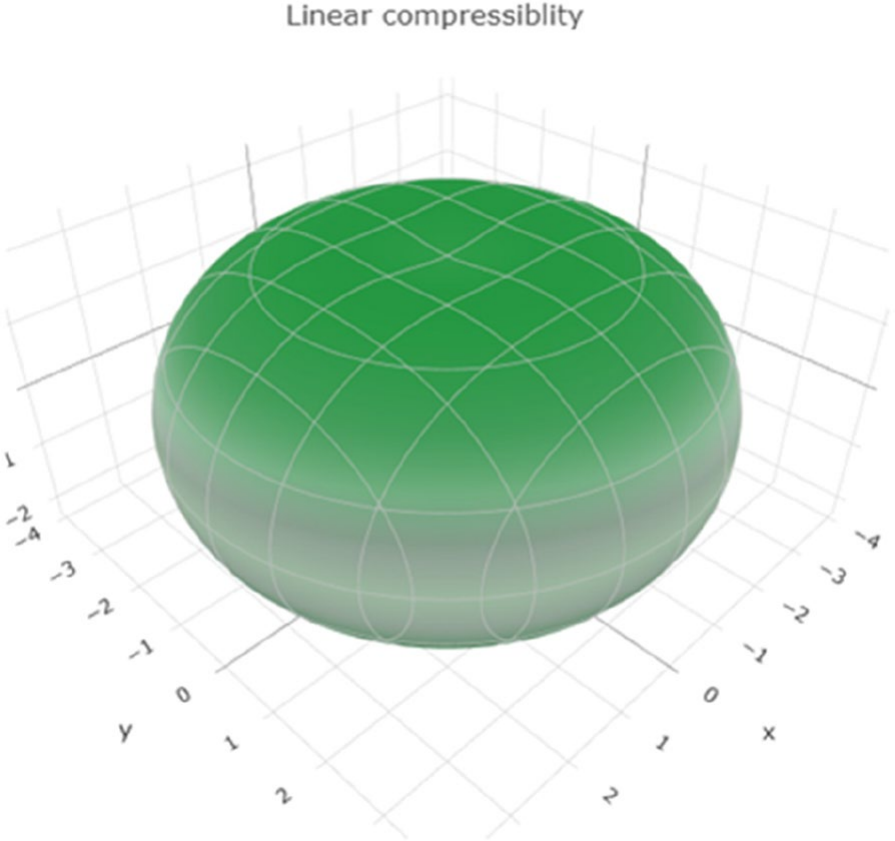
3D representation of linear compressibility of Ti metal

Linear compressibility measures the extent to which a material compresses or expands under a given stress. The range of values in Fig. 9 reveals a 3D representation of the linear compressibility. Figure S2 shows a 2D representation of the linear compressibility in the xy, xz, and yz planes, indicating the range of linear compressibility for titanium. Higher values suggest that titanium tends to compress or expand under stress.

**Fig. 10:**
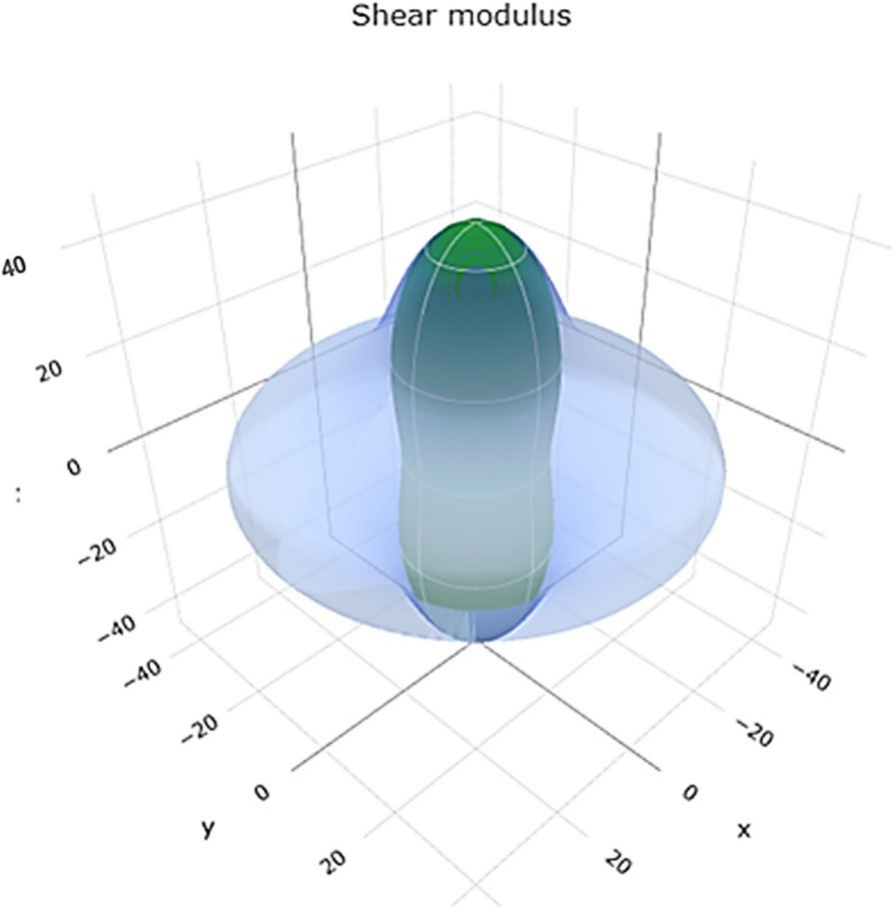
3D representation of shear modulus of Ti metal

The shear modulus represents the material's resistance to shear deformation. The range of values in Fig. 10 reveals a 3D representation of the shear modulus. Figure S3 shows a 2D representation of the shear modulus in the xy, xz, and yz planes, suggesting a range of shear resistance for Ti. Higher shear modulus values indicate better resistance to shear forces.

**Fig. 11:**
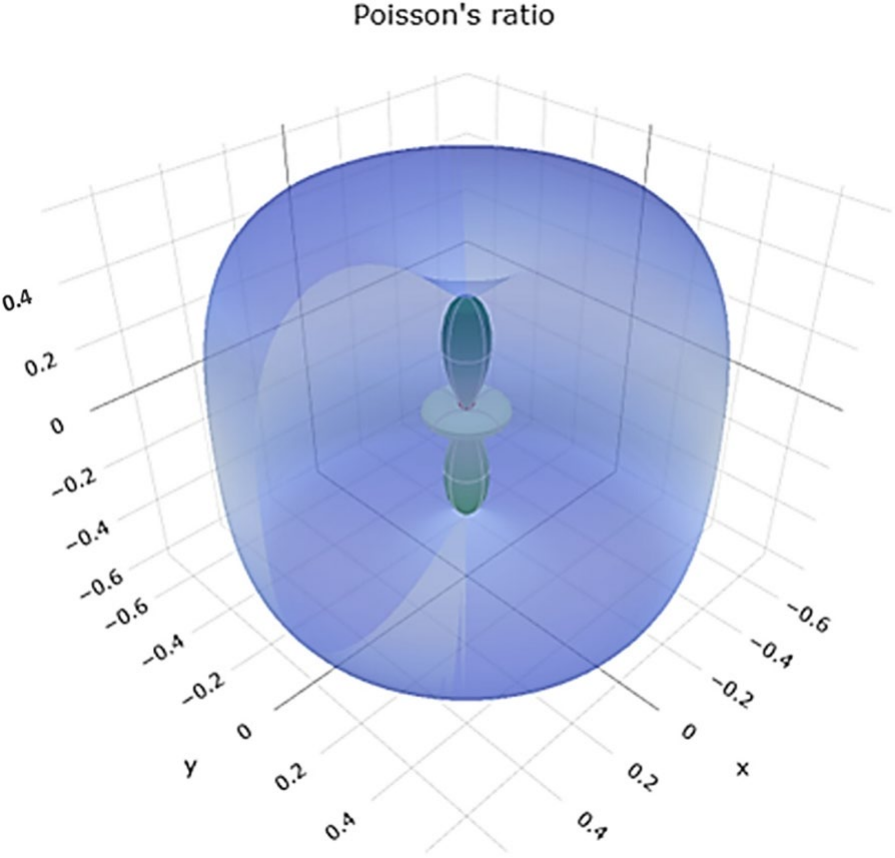
3D representation of Poisson's ratio of Ti metal

Poisson's ratio describes the ratio of lateral strain to axial strain in a material. Negative Poisson's ratio values are uncommon and may indicate unusual behavior. The range of Poisson's ratio values in Fig. 11 reveals a 3D representation. Figure S4 reveals a 2D representation in the xy, xz, and yz planes, suggesting that titanium can exhibit compressible and expandable behavior under different conditions.

Considering the variations in the elastic moduli, titanium demonstrates a wide range of mechanical properties. Depending on the specific conditions, it can exhibit a broad range of stiffness, linear compressibility, shear resistance, and Poisson's ratio. This versatility can be advantageous for dental implants, allowing for customization based on individual patient needs.

However, it is essential to note that the anisotropy of the material also plays a significant role. Table 4 lists the anisotropy values, which indicate that the properties of titanium vary depending on the direction of deformation. Anisotropy implies that material properties may differ along different axes, which should be considered when designing dental implants.

## Mechanical properties of Ti–Al-2 V alloy

### Stiffness matrix of Ti–Al-2 V alloy

The stiffness matrix in Table 5 represents the coefficients in gigapascals (GPa) for the various components. Each coefficient represents the relationship between stress and strain in different directions. The diagonal elements of the matrix represent the stiffness in the principal directions, whereas the off-diagonal elements represent the shear stiffness. The diagonal elements (331.7023, 331.9282, and 298.97305) indicate the main directions' stiffness. These values suggest that Ti–Al-2 V has relatively high stiffness, which is desirable for dental implants because it provides structural support and prevents excessive deformation under load. The off-diagonal elements (-0.0064, -0.00608, -0.00878, -0.00623, and -0.00605) represent the shear stiffness. These values are relatively small, indicating that Ti–Al-2 V has low shear stiffness. This property can benefit dental implants, allowing flexibility and reducing the risk of stress concentration and potential fracture. In comparison with titanium in general, titanium has a relatively lower stiffness than Ti–Al-2 V. This lower stiffness allows for better load distribution and minimizes stress transfer to the surrounding bone, which can be advantageous for dental implants.

**Table 5:** Stiffness matrix (coefficients in GPa) of Ti–Al-2 V alloy

331.7023	43.2861	69.00067	0.07172	-0.0064	-0.00623
43.2861	331.9282	69.11027	0.07172	-0.00608	-0.00605
69.00067	69.11027	298.97305	0.07142	-0.00878	-0.00238
0.07172	0.07172	0.07142	108.78275	-0.00343	0.0004
-0.0064	-0.00608	-0.00878	-0.00343	108.78665	0.00043
-0.00623	-0.00605	-0.00238	0.0004	0.00043	57.12235

## Average properties of Ti–Al-2 V alloy

To evaluate the suitability of Ti–Al-2 V for dental implants and compare it with titanium, we can use the average properties derived from Table 6. The average properties were calculated using three averaging schemes: Voigt, Reuss, and Hill. These schemes provide different estimates of the overall mechanical behavior of the material.

**Table 6:** Average properties of Ti–Al-2 V alloy

Averaging scheme	Bulk modulus	Young's modulus	Shear modulus	Poisson's ratio
Voigt	*K*_V_ = 147.27 GPa	*E*_V_ = 258.45 GPa	*G*_V_ = 107.02 GPa	*ν*_V_ = 0.2075
Reuss	*K*_R_ = 147.25 GPa	*E*_R_ = 239.03 GPa	*G*_R_ = 97.211 GPa	*ν*_R_ = 0.22945
Hill	*K*_H_ = 147.26 GPa	*E*_H_ = 248.83 GPa	*G*_H_ = 102.12 GPa	*ν*_H_ = 0.21838

The bulk modulus represents the resistance of a material to volume changes under pressure. A higher bulk modulus indicates greater stiffness, which is desirable for dental implants to resist deformation. Young's modulus measures the stiffness of a material in response to tensile or compressive forces. A higher Young's modulus signifies greater stiffness, which is advantageous for providing structural support for dental implants. The shear modulus indicates the resistance of a material to shear deformation. A higher shear modulus implies greater rigidity, which can contribute to the stability and strength of dental implants. Poisson's ratio describes the lateral contraction of a material when subjected to axial strain. A lower Poisson's ratio indicates less lateral deformation, which can be beneficial for minimizing the stress concentration in dental implants.

The values given in Table 7 represent the stiffness matrix for Ti–Al-2 V. These eigenvalues indicate the stiffness in different directions for Ti–Al-2 V. However, in general, titanium typically exhibits lower stiffness than Ti–Al-2 V. Lower stiffness is generally advantageous for dental implants, allowing for better load distribution and reducing stress transfer to the surrounding bone. This can help minimize the risk of bone resorption and implant failure.

**Table 7:** Eigenvalues of the stiffness matrix of Ti–Al-2 V alloy

λ_1_	λ_2_	λ_3_	λ_4_	λ_5_	λ_6_
57.122 GPa	108.78 GPa	108.79 GPa	232.22 GPa	288.53 GPa	441.85 GPa

It is important to note that the mechanical properties, including eigenvalues, are just one aspect to consider when evaluating the suitability of a material for dental implants. Factors such as biocompatibility, corrosion resistance, and osseointegration potential also play crucial roles in material selection.

## Elastic moduli of Ti–Al-2 V alloy

**Fig. 12:**
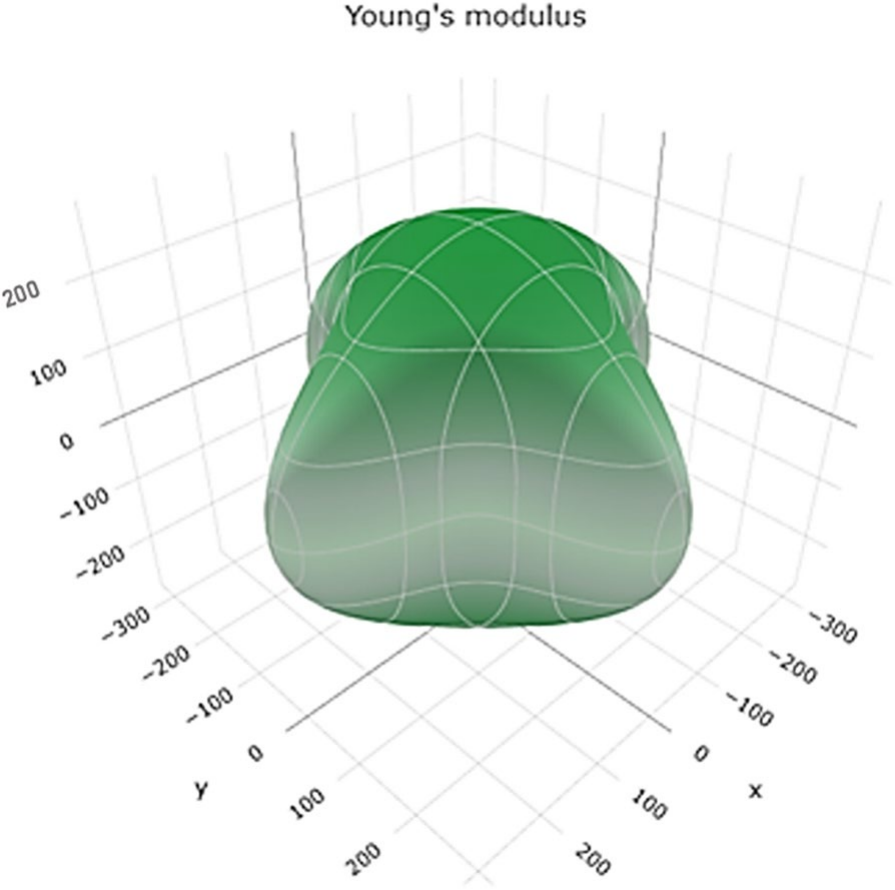
3D representation of young's modulus of Ti–Al-2 V alloy

**Fig. 13:**
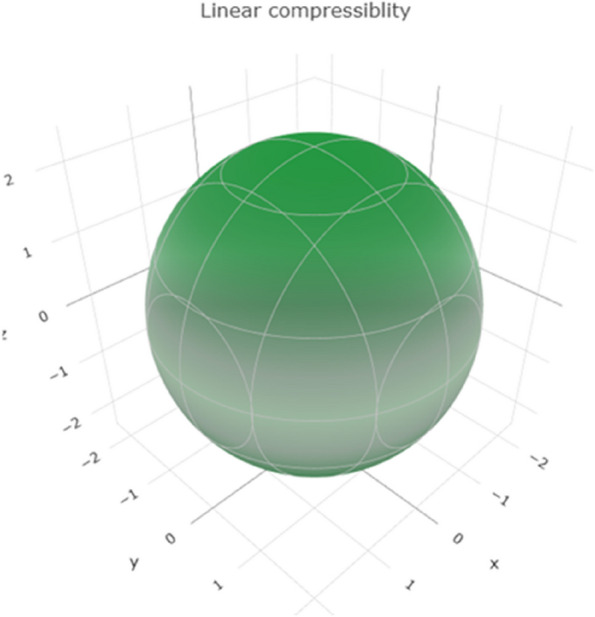
3D representation of linear compressibility of Ti–Al-2 V alloy

**Fig. 14:**
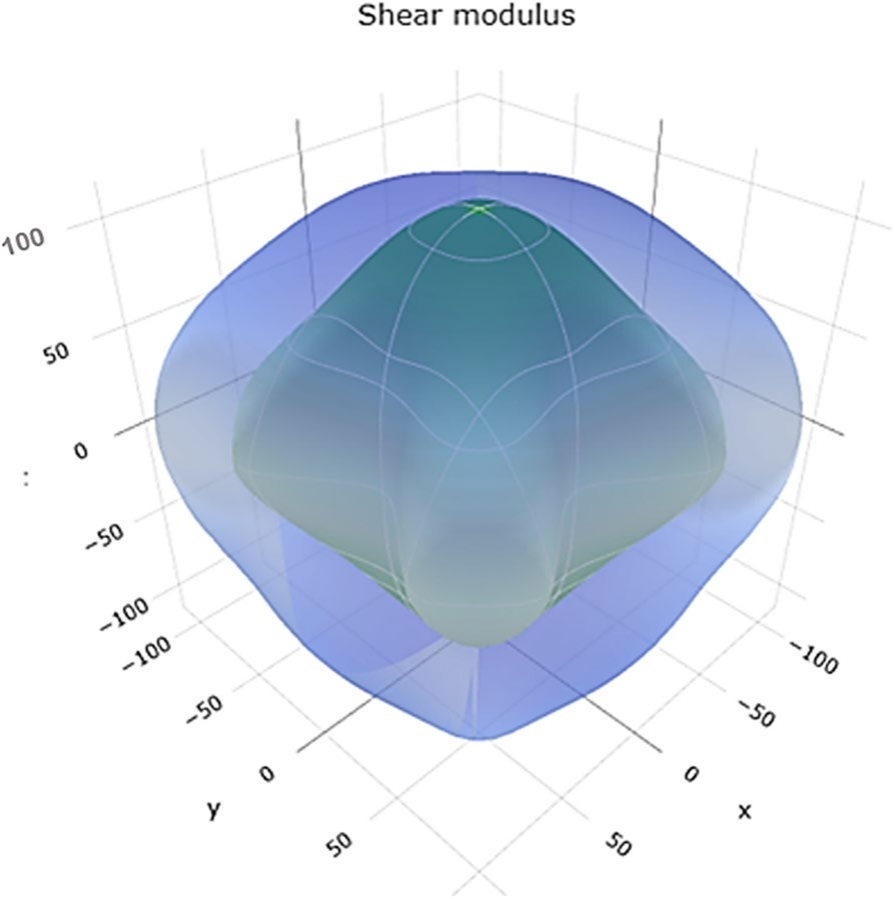
3D representation of shear modulus of Ti–Al-2 V alloy

**Fig. 15:**
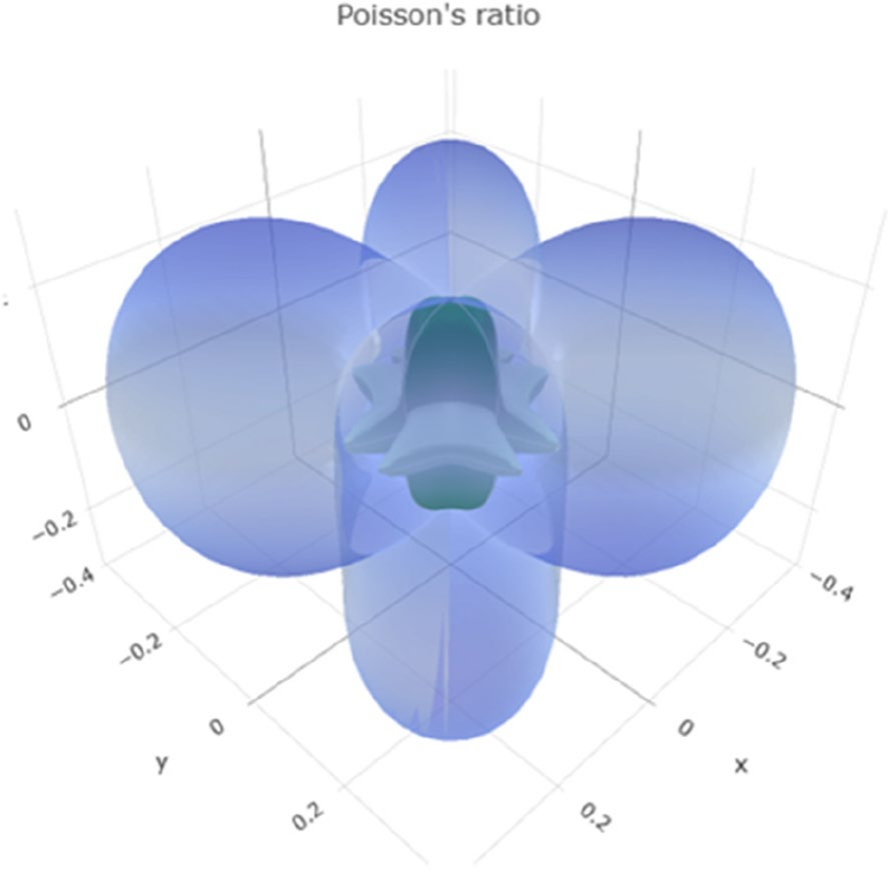
3D representation of Poisson's ratio of Ti–Al-2 V alloy

To assess the suitability of Ti–Al-2 V for dental implants and compare it with titanium, we analyzed the variations in the elastic moduli provided in Table 8. The variations in the elastic moduli give information on the anisotropy and directional properties of the material (Figs. 12, 13, 14 and 15 for 3D and Figures S5-S8 for 2D, respectively.

**Table 8:** Variations in the elastic moduli of the Ti–Al-2 V alloy

	**Young's modulus**		**Linear compressibility**		**Shear modulus**		**Poisson's ratio**		
	***E*** **min**	***E*** **max**	**β** **min**	**β** **max**	***G*** **min**	***G*** **max**	**ν** **min**	**ν** **max**	
Value	171.42 GPa	313.59 GPa	2.2393 TPa^–1^	2.3097 TPa^–1^	57.122 GPa	144.26 GPa	0.059882	0.50054	Value
Anisotropy	1.829		1.0314		2.526		8.3587		Anisotropy
Axis	0.7072	0	-0.1076	0.0031	-1	0.7071	0.5202	-0.7071	Axis
	0.707	1	0.9937	-0.0318	0	0.7071	0.5211	0.7072	
	-0.0002	0.001	0.0319	0.9995	0	0.0001	-0.6766	0.0034	
					0	-0.7071	0.4791	-0.7072	Second axis
					-1	0.7071	0.4778	-0.7071	
					0	0	0.7363	-0.0002

The minimum Young's modulus (E_min_) is 171.42 GPa, and the maximum Young's modulus (E_max_) is 313.59 GPa. This indicates that Ti–Al-2 V has anisotropic behavior, with the stiffness varying depending on the loading direction. The anisotropy factor was approximately 1.829, suggesting significant variations in stiffness in different directions.

The minimum linear compressibility (β_min_) is 2.2393 TPa^(^−1^), and the maximum linear compressibility (β_max_) is 2.3097 TPa^(^−1^). This parameter characterizes the response of the material to volumetric strains. The anisotropy factor was approximately 1.0314, indicating slight variations in compressibility in different directions.

The minimum shear modulus (G_min_) is 57.122 GPa, and the maximum shear modulus (G_max_) is 144.26 GPa. Ti–Al-2 V exhibited considerable variations in shear stiffness depending on the loading direction, with an anisotropy factor of approximately 2.526.

The minimum Poisson's ratio (ν_min_) was 0.059882, and the maximum Poisson's ratio (ν_max_) was 0.50054. Poisson's ratio indicates the lateral contraction of a material under axial strain. Ti–Al-2 V exhibited significant anisotropy in Poisson's ratio with an anisotropy factor of approximately 8.3587, implying a substantial variation in lateral deformation in different directions.

In general, titanium is known to have relatively lower anisotropy in its elastic properties compared to Ti–Al-2 V. Titanium is commonly used for dental implants due to its favorable mechanical properties, including biocompatibility and corrosion resistance.

## Mechanical properties of zirconia

### Stiffness matrix of zirconia

To assess the suitability of zirconia for dental implants and compare it with titanium, we can analyze the stiffness matrix provided in Table 9. These values indicate that zirconia has high stiffness, which is desirable for dental implants as it allows for structural support and resistance to deformation. The off-diagonal elements represent the shear stiffness. These values suggest that zirconia has a relatively high shear stiffness, contributing to its strength and stability. Compared with Titanium, Zirconia generally has higher stiffness values compared to titanium. This indicates that zirconia is a stiffer material that can provide greater structural support and resistance to deformation in dental implant applications.

**Table 9:** Stiffness matrix (coefficients in GPa) of zirconia

701.26	95.536	95.536	0	0	0
95.536	701.26	95.536	0	0	0
95.536	95.536	701.26	0	0	0
0	0	0	131.13	0	0
0	0	0	0	131.13	0
0	0	0	0	0	131.13

## Average properties of zirconia

To evaluate the suitability of zirconia for dental implants and compare it with titanium, we can utilize the average properties provided in Table 10. The bulk modulus represents the material's resistance to volume changes under pressure. Zirconia exhibited a relatively high average bulk modulus, indicating good stiffness and resistance to compression. Young's modulus measures the material's stiffness in response to tensile or compressive forces. Zirconia exhibited a high average Young's modulus, indicating excellent structural support and rigidity. The shear modulus reflects the resistance of the material to shear deformation. Zirconia has a relatively high average shear modulus, contributing to its strength and stability. Poisson's ratio characterizes the lateral contraction of a material under axial strain. Zirconia demonstrated average Poisson's ratios within a reasonable range, indicating limited lateral deformation. Zirconia generally has higher average values of bulk modulus, Young's, and shear modulus than titanium. This suggests that zirconia is stiffer, possesses greater structural support, and is more deformation-resistant. Regarding Poisson's ratio, zirconia and titanium have similar ranges, indicating limited lateral deformation in both materials.

**Table 10:** Average properties of zirconia

Averaging scheme	Bulk modulus	Young's modulus	Shear modulus	Poisson's ratio
Voigt	*K*_V_ = 297.44 GPa	*E*_V_ = 489.79 GPa	*G*_V_ = 199.82 GPa	*ν*_V_ = 0.22555
Reuss	*K*_R_ = 297.44 GPa	*E*_R_ = 427.54 GPa	*G*_R_ = 169.6 GPa	*ν*_R_ = 0.26044
Hill	*K*_H_ = 297.44 GPa	*E*_H_ = 459.1 GPa	*G*_H_ = 184.71 GPa	*ν*_H_ = 0.24275

To evaluate the suitability of zirconia for dental implants and compare it with titanium, we can analyze the eigenvalues of the stiffness matrix provided in Table 11. Zirconia exhibited three identical eigenvalues (131.13 GPa), indicating isotropic behavior in the three principal directions. This suggests that zirconia has consistent stiffness and mechanical properties when loaded in different directions. The remaining three eigenvalues (605.72 GPa and 892.33 GPa) represent stiffness values in other directions. These higher eigenvalues indicate that zirconia may exhibit additional anisotropic behavior and varying stiffness in specific directions. In general, titanium is known to have lower stiffness than zirconia.

**Table 11:** Eigenvalues of the stiffness matrix of zirconia

λ_1_	λ_2_	λ_3_	λ_4_	λ_5_	λ_6_
131.13 GPa	131.13 GPa	131.13 GPa	605.72 GPa	605.72 GPa	892.33 GPa

## Elastic moduli of zirconia

**Fig. 16:**
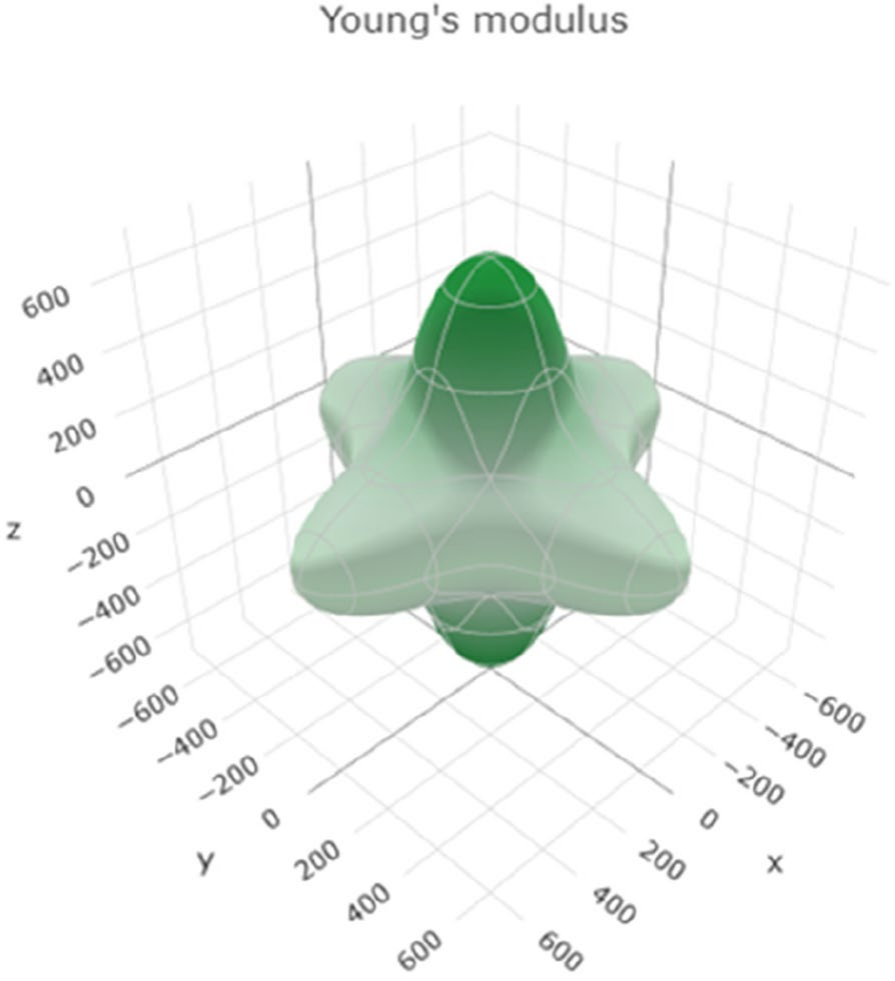
3D representation of Young's modulus of zirconia

**Fig. 17:**
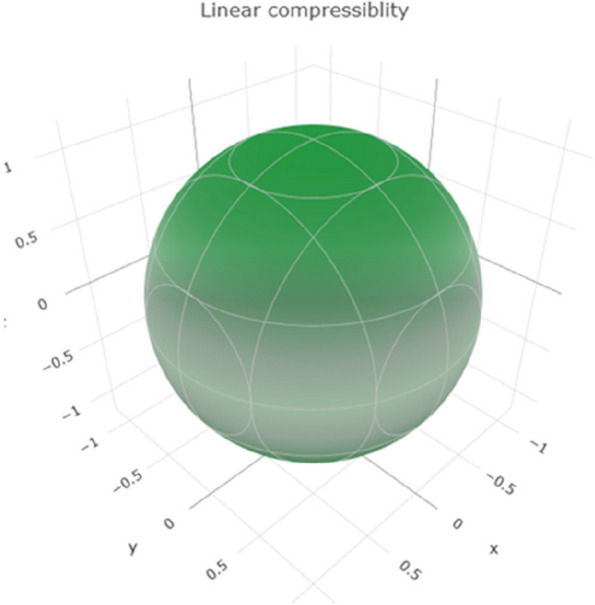
3D representation of linear compressibility of zirconia

**Fig. 18:**
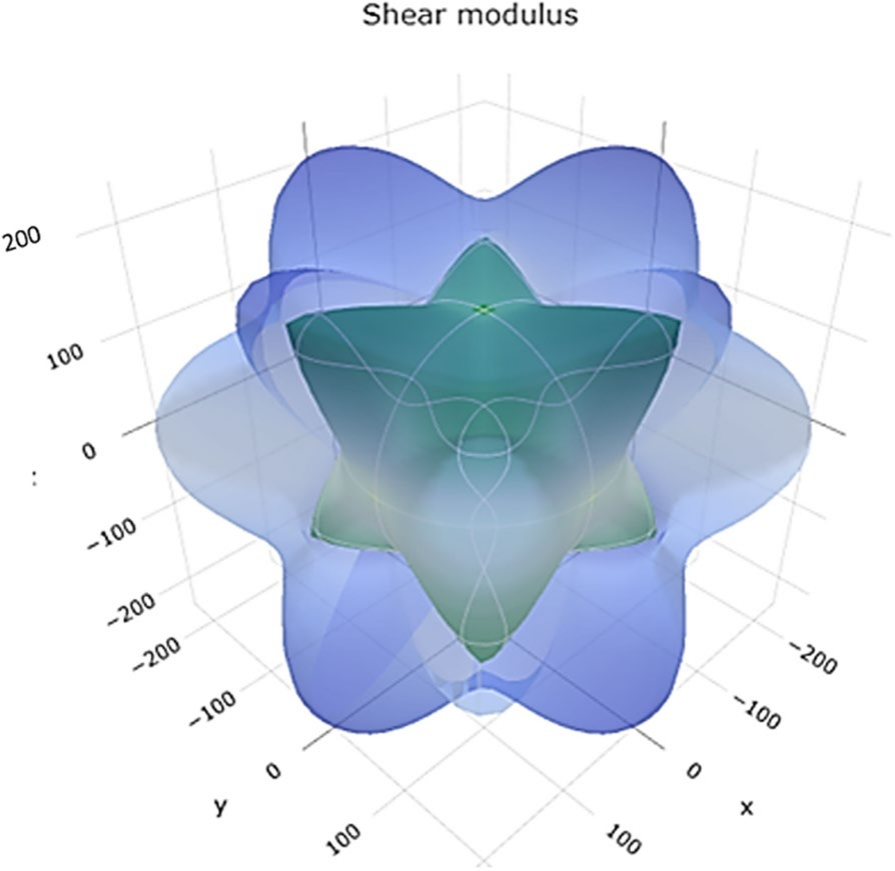
3D representation of shear modulus of zirconia

**Fig. 19:**
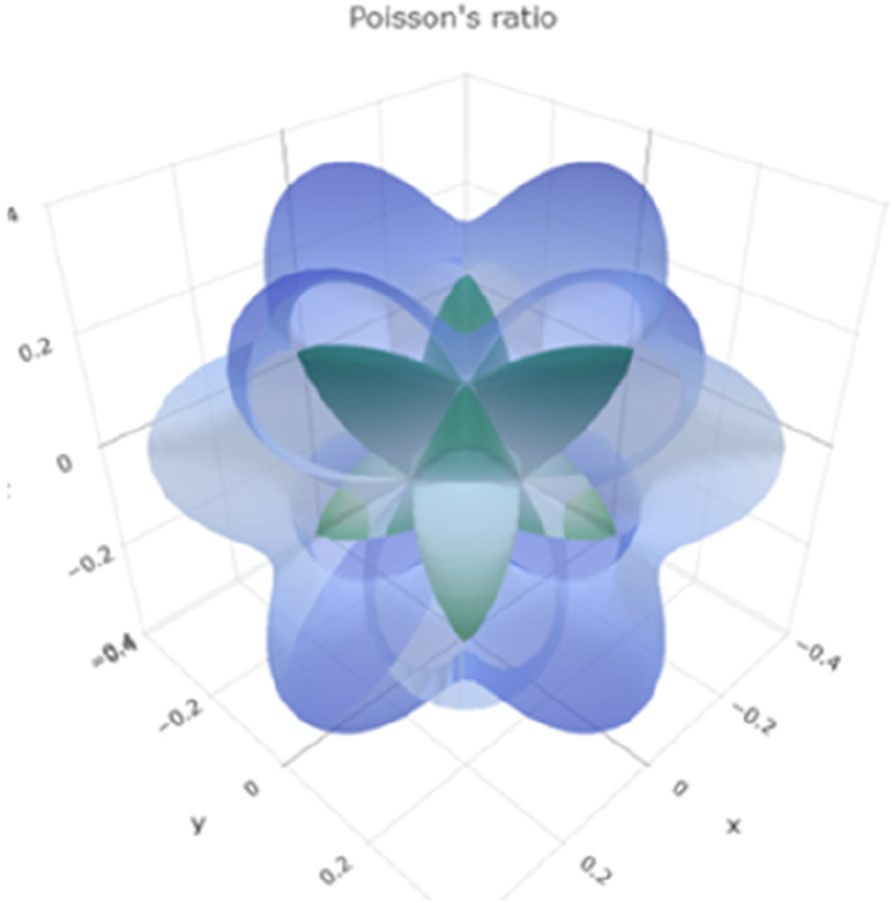
3D representation of Poisson's ratio of zirconia

To assess the suitability of zirconia for dental implants and compare it with titanium, we analyzed the variations in the elastic moduli provided in Table 12. These variations offer information about the range and anisotropy of the material's elastic properties (Figs. 16, 17, 18 and 19 for 3D and S9-S12 for 2D, respectively.

**Table 12:** Variations in the elastic moduli of zirconia

	**Young's modulus**		**Linear compressibility**		**Shear modulus**		**Poisson's ratio**		
	***E*** **min**	***E*** **max**	**β** **min**	**β** **max**	***G*** **min**	***G*** **max**	**ν** **min**	**ν** **max**	
Value	342.99 GPa	678.35 GPa	1.1207 TPa^–1^	1.1207 TPa^–1^	131.13 GPa	302.86 GPa	0.069175	0.49224	Value
Anisotropy	1.978		1.0000		2.31		7.1158		Anisotropy
Axis	0.5774 0.5773 -0.5774	1.0000 0.0000 0.0000	0.2053 0.7663 0.6088	-0.2500 0.9330 -0.2588	0.0000 0.0000 1.0000	0.7071 0.0001 -0.7071	0.7071 -0.0000 0.7071	0.7071 -0.0002 0.7071	Axis
					-0.7660 0.6428 0.0000	-0.7071 -0.0002 -0.7071	0.0000 1.0000 0.0000	0.7071 -0.0005 -0.7071	Second axis

Young's zirconia modulus variations are given as E_min_ = 342.99 GPa and E_max_ = 678.35 GPa. This indicates that zirconia has a range of Young's modulus values, with a minimum of 342.99 GPa and a maximum of 678.35 GPa. The higher range of Young's moduli suggests that zirconia can exhibit varying degrees of stiffness, which is advantageous for dental implants that require specific mechanical properties.

The variations in linear compressibility for zirconia are given by β_min_ = 1.1207 TPa^–1^ and β_max_ = 1.1207 TPa^–1^. Linear compressibility measures the change in volume per unit of applied pressure. The constant values for linear compressibility suggest that zirconia exhibits isotropic behavior regarding volume changes under pressure.

The variations in shear modulus for zirconia are given as G_min_ = 131.13 GPa and G_max_ = 302.86 GPa. This indicates that zirconia can exhibit varying shear moduli within this range. The higher shear modulus values signify the material's resistance to shear deformation and ability to withstand the applied forces.

Poisson's zirconia ratio variations are ν_min_ = 0.069175 and ν_max_ = 0.49224. Poisson's ratio represents the ratio of lateral strain to axial strain. The range of Poisson's ratios suggests that zirconia can exhibit different degrees of lateral deformation when subjected to axial strain. Zirconia generally has higher Young's and shear modulus than titanium, indicating superior stiffness and mechanical strength. Table 13 demonstrates the comprehensive analysis of the three materials.

**Table 13:** The comprehensive analysis of the materials used in the study

Property/Characteristic	Ti Metal	Ti–Al-2 V Alloy	Zirconia
Crystal Structure	Simple Hexagonal (Space Group: *P*63/MMC)	Simple Triclinic (Space Group: P1)	Simple Cubic (Space Group: FM-3 M)
Atomic Arrangement	Ti atoms at simple cubic corner lattice points; hexagonal lattice in the basal plane	Ti atoms at simple cubic corner lattice points, Al atoms at center, V atoms between Ti atoms along c-axis	Zn atoms at simple cubic corner lattice points, forming connections with O atoms
Electronic Band Structure	Complete bandgap; Stable electronic structure with well-organized arrangement	Complete bandgap; Presence of imaginary frequencies indicates instability	Complete bandgap; Wider energy range suggests potential complex phonon structure
Dynamic Stability	Dynamically stable with absence of imaginary frequencies within specified energy range	Presence of imaginary frequencies indicating instability	Presence of imaginary frequencies indicating potential instability
Fermi Surface	Well-defined, nearly complete drum-shaped Fermi surface indicating good mechanical stability and interatomic bonding	-	-
Stiffness Matrix	Diagonal: 198.14 GPa, Off-diagonal: -	Diagonal: 331.70–331.93 GPa, Off-diagonal: -0.0064 to -0.00605	Diagonal: 131.13 GPa, Off-diagonal: Relatively High
Average Properties	Bulk Modulus: 101–104 GPa, Young's Modulus: 64–88 GPa, Shear Modulus: 23–32 GPa, Poisson's Ratio: 0.358–0.394	Bulk Modulus: Higher, Young's Modulus: Higher, Shear Modulus: Higher, Poisson's Ratio: Similar Range	Bulk Modulus: Relatively High, Young's Modulus: High, Shear Modulus: Relatively High, Poisson's Ratio: Limited Lateral Deformation
Eigenvalues	Eigenvalues: 13.485 GPa, 26.969 GPa, 44.526 GPa (Stiffness in Different Directions)	Eigenvalues: 131.13 GPa (Identical in Principal Directions), 605.72 GPa, 892.33 GPa (Other Directions)	Eigenvalues: 131.13 GPa (Isotropic in Principal Directions), 605.72 GPa, 892.33 GPa (Other Directions)
Elastic Moduli Variations	Young's Modulus Range: 64–88 GPa, Linear Compressibility: Range, Shear Modulus Range: 23–32 GPa, Poisson's Ratio Range: 0.358–0.394	Young's Modulus Range: 171.42 GPa to 313.59 GPa, Linear Compressibility: Constant, Shear Modulus Range: 57.122 GPa to 144.26 GPa, Poisson's Ratio Range: 0.059882 to 0.50054	Young's Modulus Range: 342.99 GPa to 678.35 GPa, Linear Compressibility: Constant, Shear Modulus Range: 131.13 GPa to 302.86 GPa, Poisson's Ratio Range: 0.069175 to 0.49224

After careful consideration, Titanium (Ti) metal is the optimal choice for dental implants due to its remarkable combination of properties. Its stable crystal structure ensures structural integrity and exhibits excellent dynamic stability, which is crucial for long-term performance. Titanium boasts balanced mechanical properties, such as high stiffness and resistance to deformation, essential for withstanding oral forces. The maintenance of dynamic stability in titanium implants is of paramount importance in ensuring their sustained efficacy and dependability over an extended period of time. Maintaining a stable implant is crucial in preserving the integrity of the crystal lattice, hence preventing any potential structural instabilities, phase transitions, or lattice defects that may affect the implant's mechanical properties and overall functionality.

Titanium demonstrates exceptional resistance to deformation when exposed to axial stresses. The ability to endure chewing pressures is a desirable characteristic for dental implants. Titanium exhibits advantageous stiffness characteristics for dental implants, as inferred from the eigenvalues. This implies that titanium possesses favorable features that make it highly capable of enduring the mechanical stresses imposed on dental implants during mastication and other oral functions. Moreover, it has a proven track record in dental implant applications, emphasizing its reliability.

Zirconia demonstrates remarkable biocompatibility, rendering it suitable for use with oral tissues and reducing the likelihood of adverse responses or inflammation. Moreover, zirconia has exceptional strength and durability, which can be comparable to that of titanium implants. The natural white color of zirconia contributes to its outstanding aesthetic qualities, allowing it to harmonize effortlessly with adjacent teeth and enhance the overall visual appeal by creating a more realistic and attractive look. In general, zirconia exhibits higher stiffness values than titanium. This finding suggests that zirconia possesses a higher rigidity level, enabling it to offer enhanced structural reinforcement and increased resistance to deformation when utilized in dental implant scenarios. Zirconia has a comparatively elevated mean shear modulus, improving its mechanical strength and structural stability. Higher eigenvalues suggest that zirconia has the potential to display extra anisotropic characteristics and variable stiffness in particular orientations.

While Ti–Al-2 V Alloy and Zirconia have distinct features, titanium's well-rounded properties and its established history make it the preferred material, ensuring durability, stability, and biocompatibility in dental implant procedures.

## Conclusions

In conclusion, using Density Functional Theory (DFT) has provided valuable insights into dental implant materials' mechanical properties and structural stability. Titanium (Ti) has been highlighted as an excellent choice for dental implants due to its biocompatibility, osseointegration ability, exceptional strength-to-weight ratio, resistance to fractures and corrosion, lightweight nature, hypoallergenic properties, and promotion of tissue adhesion. Using DFT, biocompatibility and osseointegration can be predicted and measured by simulating the interactions between dental implant materials and biological molecules or tissues at the atomic level. DFT calculations can assess the electronic structure and energetics of these interactions, providing insights into the stability of implant surfaces, their reactivity with surrounding biomolecules, and the likelihood of triggering immune responses. By analyzing binding energies, charge transfer, and electronic properties, DFT enables scientists to predict the compatibility of implant materials with the human body and their ability to integrate seamlessly with bone tissue, crucial factors in ensuring successful dental implantation proceduresAlternative materials like titanium alloys and zirconia have also been considered. DFT allows material behavior simulation in different physiological conditions and mechanical stresses, aiding in material selection. It provides insights into dental implant materials' mechanical behavior, structural stability, and fatigue resistance, contributing to their longevity and success. Dental implant materials can be evaluated for parameters like tensile strength, yield strength, ductility, elastic modulus, hardness, fatigue resistance, and corrosion resistance. DFT has significantly advanced the understanding and development of dental implant materials, leading to more durable and biocompatible options.

### Supplementary Information

**Additional file 1: Figure S1. **2D representation of Young's modulus of Ti metal in xy, xz and yz plane. **Figure S2.** 2D representation of linear compressibility of Ti metal in xy, xz and yz plane. **Figure S3.** 2D representation of Shear modulus of Ti metal in xy, xz and yz plane. **Figure S4.** 2D representation of Poisson's ratio of Ti metal in xy, xz and yz plane. **Figure S5.** 2D representation of Youngs’s modulus of TiAl_2_V in xy, xz and yz plane. **Figure S6.** 2D representation of linear compressibility of TiAl_2_V in xy, xz and yz plane. **Figure S7.** 2D representation of Shear modulus of TiAl_2_V in xy, xz and yz plane. **Figure S8.** 2D representation of Poisson’s ratio of TiAl_2_V in xy, xz and yz plane. **Figure S9.** 2D representation of Youngs’s modulus of Zirconia in xy, xz and yz plane. **Figure S10.** 2D representation of linear compressibility of Zirconia in xy, xz and yz plane. **Figure S11. **2D representation of Shear modulus of Zirconia in xy, xz and yz plane. **Figure S12.** 2D representation of Poisson’s ratio of Zirconia in xy, xz and yz plane.

## Data Availability

The data supporting this study's findings are available from the corresponding author upon reasonable request.

## Abbreviations

Ti–Al-2 V: Titanium alloys
Ti: Titanium
DFT: Density Functional Theory
CASTEP: Cambridge Serial Total Energy Package
PBE: Perdew-Burke-Ernzerhof
LBFGS: Limited-memory Broyden–Fletcher–Goldfarb–Shanno
BZ: Brillouin zone
GPa: Gigapascals
