# Supplementary material for: Application of density functional theory for evaluating the mechanical properties and structural stability of dental implant materials
Source: BMC Oral Health. 2023 Dec 1;23:958. doi: 10.1186/s12903-023-03691-8 (PMC10693112; doi:10.1186/s12903-023-03691-8)
Supplement: Supplementary file 1 — Additional file 1: Figure S1. 2D representation of Young's modulus of Ti metal in xy, xz and yz plane. Figure S2. 2D representation of linear compressibility of Ti metal in xy, xz and yz plane. Figure S3. 2D representation of Shear modulus of Ti metal in xy, xz and yz plane. Figure S4. 2D representation of Poisson's ratio of Ti metal in xy, xz and yz plane. Figure S5. 2D representation of Youngs’s modulus of TiAl2V in xy, xz and yz plane. Figure S6. 2D representation of linear compressibility of TiAl2V in xy, xz and yz plane. Figure S7. 2D representation of Shear modulus of TiAl2V in xy, xz and yz plane. Figure S8. 2D representation of Poisson’s ratio of TiAl2V in xy, xz and yz plane. Figure S9. 2D representation of Youngs’s modulus of Zirconia in xy, xz and yz plane. Figure S10. 2D representation of linear compressibility of Zirconia in xy, xz and yz plane. Figure S11. 2D representation of Shear modulus of Zirconia in xy, xz and yz plane. Figure S12. 2D representation of Poisson’s ratio of Zirconia in xy, xz and yz plane. [file 12903_2023_3691_MOESM1_ESM.docx]

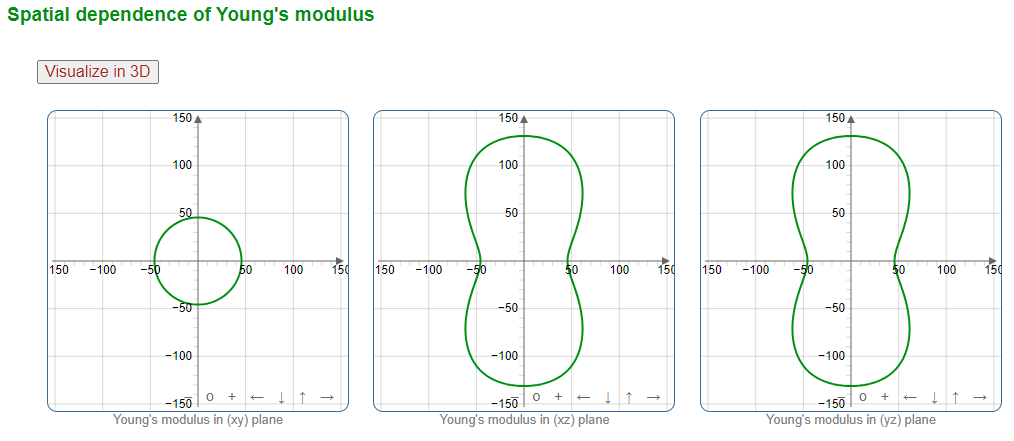


Figure S1: 2D representation of Young's modulus of Ti metal in xy, xz and yz plane.


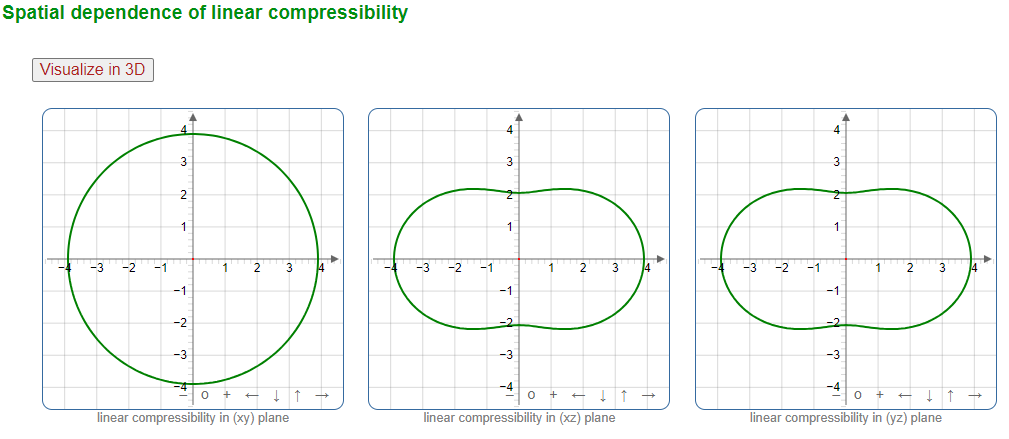


Figure S2: 2D representation of linear compressibility of Ti metal in xy, xz and yz plane.


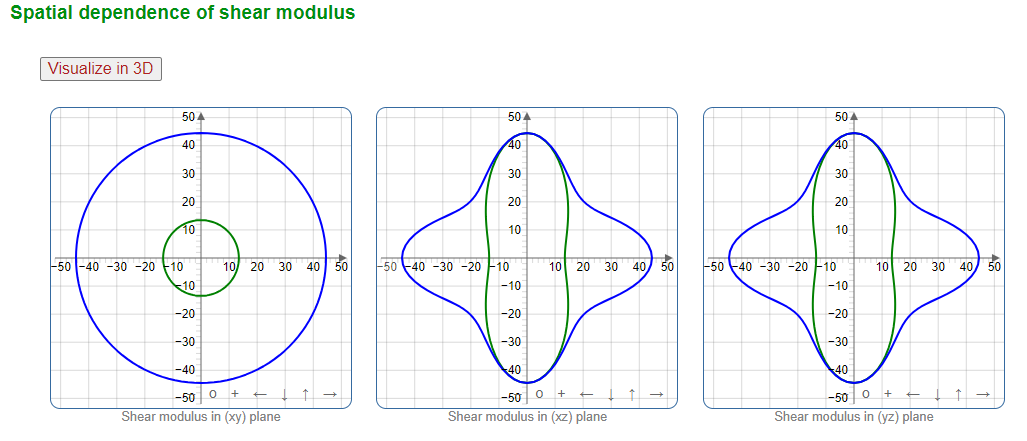


Figure S3: 2D representation of Shear modulus of Ti metal in xy, xz and yz plane.


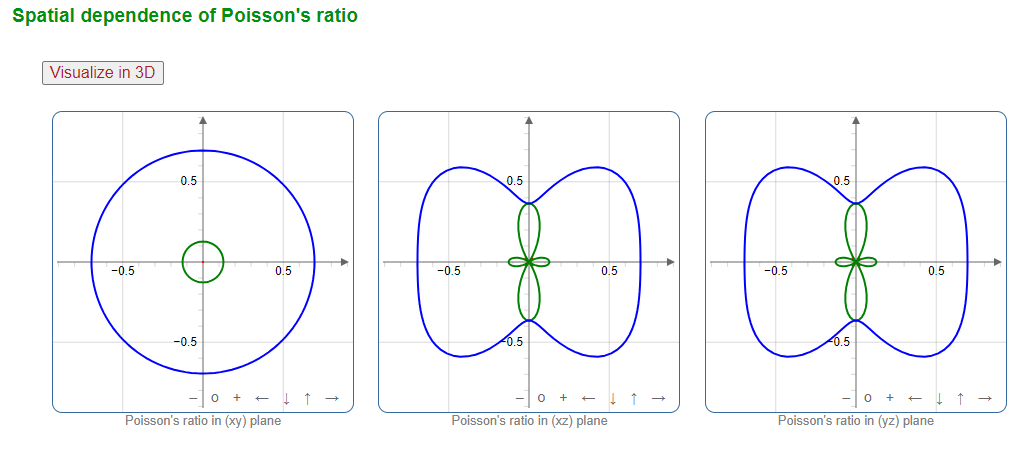


Figure S4: 2D representation of Poisson's ratio of Ti metal in xy, xz and yz plane.

Alloy


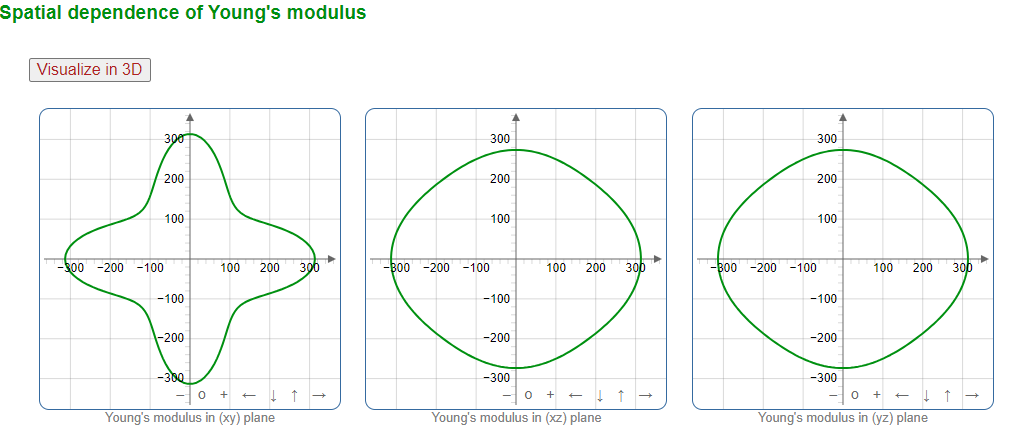


Figure S5: 2D representation of Youngs’s modulus of TiAl_2_V in xy, xz and yz plane.


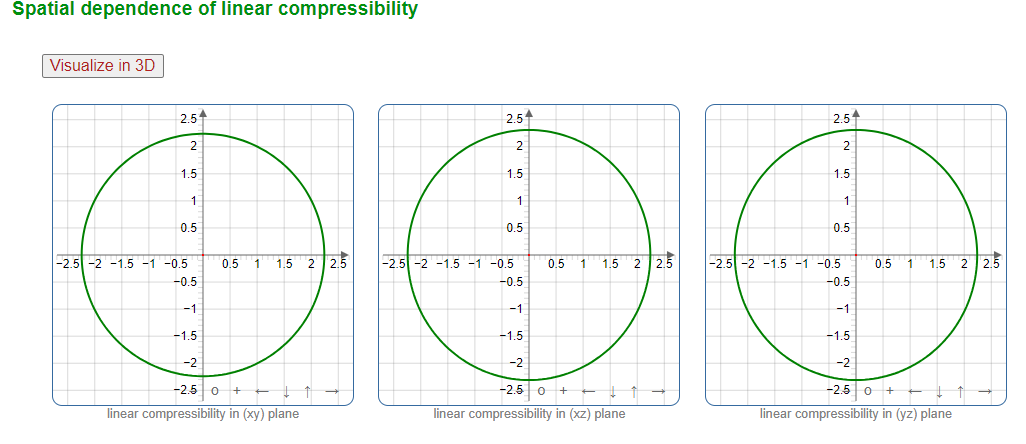


Figure S6: 2D representation of linear compressibility of TiAl_2_V in xy, xz and yz plane.


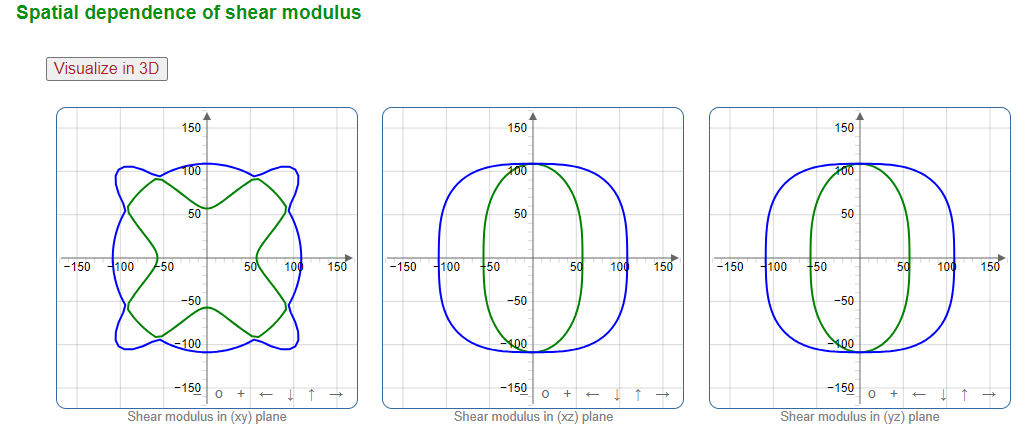


Figure S7: 2D representation of Shear modulus of TiAl_2_V in xy, xz and yz plane.


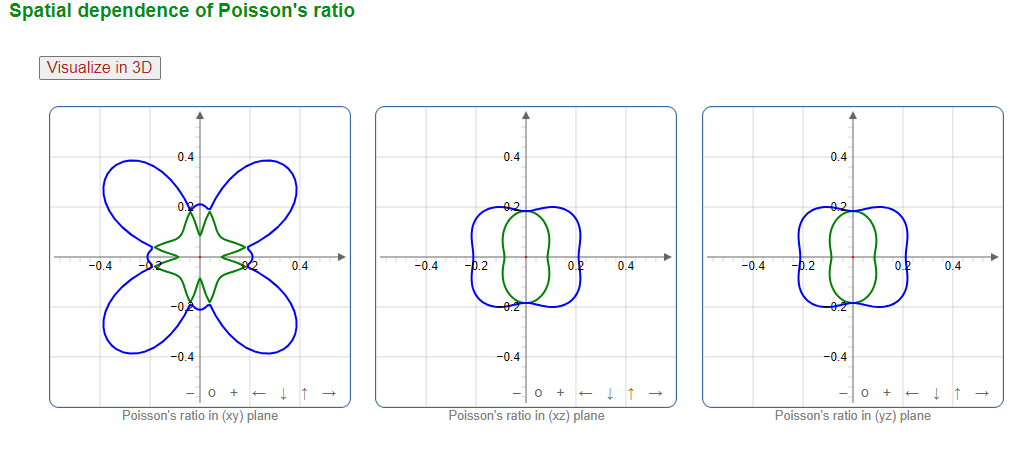


Figure S8: 2D representation of Poisson’s ratio of TiAl_2_V in xy, xz and yz plane.


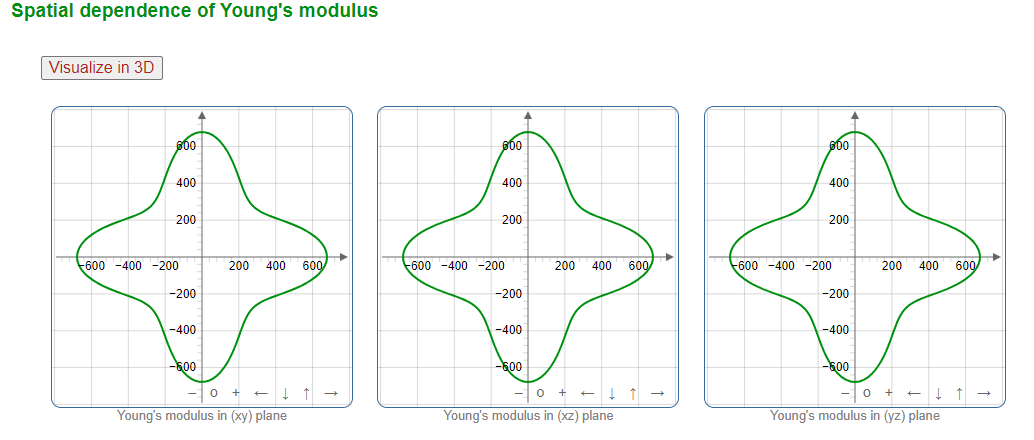


Figure S9: 2D representation of Youngs’s modulus of Zirconia in xy, xz and yz plane.


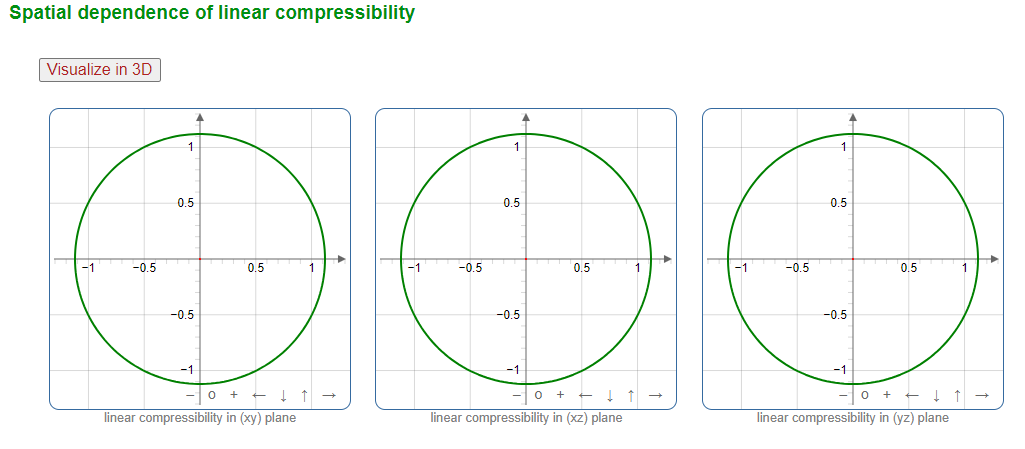


Figure S10: 2D representation of linear compressibility of Zirconia in xy, xz and yz plane.


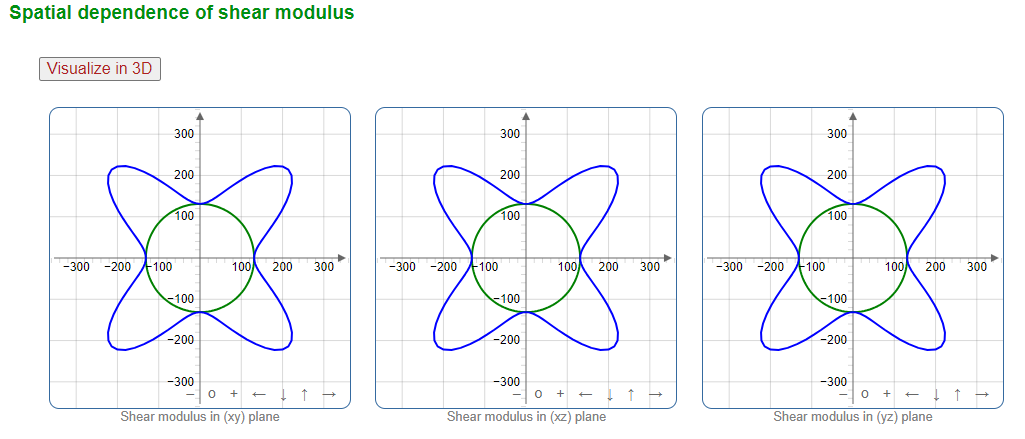


Figure S11: 2D representation of Shear modulus of Zirconia in xy, xz and yz plane.


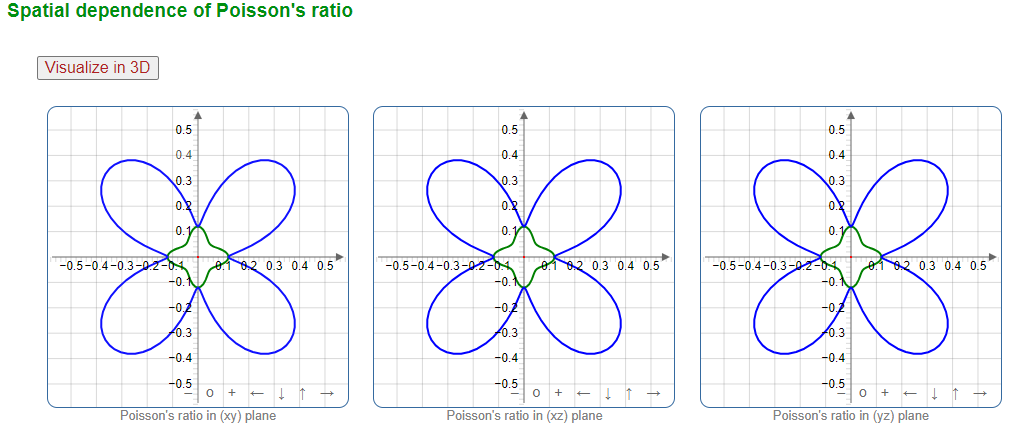


Figure S12: 2D representation of Poisson’s ratio of Zirconia in xy, xz and yz plane.
